# Cell physiology and molecular mechanism of anion transport by erythrocyte band 3/AE1

**DOI:** 10.1152/ajpcell.00275.2021

**Published:** 2021-10-20

**Authors:** Michael L. Jennings

**Affiliations:** Department of Physiology and Cell Biology, University of Arkansas for Medical Sciences, Little Rock, Arkansas

**Keywords:** band 3, bicarbonate, chloride, erythrocyte, transport

## Abstract

The major transmembrane protein of the red blood cell, known as band 3, AE1, and SLC4A1, has two main functions: *1*) catalysis of Cl^−^/$\mathrm{HC}O_{3}^{-}$ exchange, one of the steps in CO_2_ excretion, and *2*) anchoring the membrane skeleton. This review summarizes the 150-year history of research on red cell anion transport and band 3 as an experimental system for studying membrane protein structure and ion transport mechanisms. Important early findings were that red cell Cl^−^ transport is a tightly coupled 1:1 exchange and band 3 is labeled by stilbenesulfonate derivatives that inhibit anion transport. Biochemical studies showed that the protein is dimeric or tetrameric (paired dimers) and that there is one stilbenedisulfonate binding site per subunit of the dimer. Transport kinetics and inhibitor characteristics supported the idea that the transporter acts by an alternating access mechanism with intrinsic asymmetry. The sequence of band 3 cDNA provided a framework for detailed study of protein topology and amino acid residues important for transport. The identification of genetic variants produced insights into the roles of band 3 in red cell abnormalities and distal renal tubular acidosis. The publication of the membrane domain crystal structure made it possible to propose concrete molecular models of transport. Future research directions include improving our understanding of the transport mechanism at the molecular level and of the integrative relationships among band 3, hemoglobin, carbonic anhydrase, and gradients (both transmembrane and subcellular) of $\mathrm{HC}O_{3}^{-}$, Cl^−^, O_2_, CO_2_, pH, and nitric oxide (NO) metabolites during pulmonary and systemic capillary gas exchange.

## INTRODUCTION

The most abundant protein in the red blood cell membrane is known as band 3, AE1, capnophorin, and SLC4A1. Band 3 catalyzes transmembrane Cl^−^/$\mathrm{HC}O_{3}^{-}$ exchange, one of the steps in CO_2_ excretion, and is the attachment site for the membrane skeleton. This review is an attempt to summarize ∼150 years of research on red cell inorganic anion transport and the properties of band 3 as an ion transporter. The literature on band 3 and related proteins is vast, and several topics are mentioned only briefly: other transporters in the SLC4 family (1–4); nonmammalian band 3 (5, 6); and the role of band 3 in the red cell membrane skeleton (7, 8), regulation of metabolism (9–11), senescence (12–15), and malaria (8, 16, 17).

A major advance in the field was the determination of the crystal structure of the band 3 membrane domain complexed with Fab fragments (18); membrane domain crystals prepared under microgravity conditions without Fab fragments reveal a similar structure (19). Reithmeier et al. (20) have reviewed many aspects of the band 3 literature in the context of the crystal structure. To minimize repetition of the content of this excellent review, discussion of some topics is limited here, including band 3 synthesis, glycosylation, and targeting. There is also very little reference to the many models of band 3 membrane domain topology and structure that were proposed before the crystal structure was determined.

The review begins with a roughly chronological history of key early findings, from the first evidence for red cell Cl^−^ and $\mathrm{HC}O_{3}^{-}$ transport in the 1800s through the identification of band 3 as the transport protein a century later. The remainder of the article is organized by topic rather than chronology, ending with questions that remain unanswered about the molecular mechanism and cellular physiology of band 3-mediated anion transport.

## RED BLOOD CELL ANION TRANSPORT AND THE BAND 3 PROTEIN: 1867–1980

### Early Work on the Role of Red Blood Cell Cl^−^ and $\mathrm{HC}O_{3}^{-}$ Transport in CO_2_ Excretion

Three discoveries in the 1800s showed that Cl^−^ and $\mathrm{HC}O_{3}^{-}$ are transported between red blood cells and plasma. In 1867, Schmidt and Zuntz separately reported that exposure of blood to CO_2_ causes plasma $\mathrm{HC}O_{3}^{-}$ concentration ([$\mathrm{HC}O_{3}^{-}$]) to increase much more than exposure of plasma alone to CO_2_ (21). Exposing blood to CO_2_ also causes an increase in red cell volume (22) and net movement of Cl^−^ into cells (23). These effects are expected if $\mathrm{HC}O_{3}^{-}$ is formed from CO_2_ inside the cell and transported outward in exchange for Cl^−^.

Work in the early 1900s showed that graded increases in $\mathrm{PC}O_{2}$ cause increases in cellular Cl^−^ (24), and incubation of red cells in isotonic sucrose causes Cl^−^ efflux that is accelerated by small amounts of $\mathrm{HC}O_{3}^{-}$ (25), demonstrating that Cl^−^ can exchange with $\mathrm{HC}O_{3}^{-}$ in both directions. In the 1920s the steady-state distribution of Cl^−^ and $\mathrm{HC}O_{3}^{-}$ between red cells and plasma was found to be that expected for Donnan equilibrium if the membrane is much more permeable to Cl^−^ and $\mathrm{HC}O_{3}^{-}$ than to Na^+^ and K^+^ (21, 26, 27):

$${\left[{\text{Cl}}^{-}\right]}_{\text{i}}/{\left[{\text{Cl}}^{-}\right]}_{\text{o}}=\;{\left[{\text{HCO}}_{3}^{-}\right]}_{\text{i}}/{\left[{\text{HCO}}_{3}^{-}\right]}_{\text{o}}=\;{\left[{\text{OH}}^{-}\right]}_{\text{i}}/{\left[{\text{OH}}^{-}\right]}_{\text{o}}=\;{\left[{\text{H}}^{+}\right]}_{\text{o}}/{\left[{\text{H}}^{+}\right]}_{\text{i}}=\text{ r }=\text{ exp}\left(-FV_{\text{m}}/R\text{T}\right)$$

where [X]_i_ and [X]_o_ are intracellular and extracellular concentrations, r is the Donnan ratio, *V*_m_ is the membrane potential, and *F*, R, and T have their usual meanings. The Donnan ratio r is ∼0.63 at extracellular pH 7.4, 37°C (28, 29) and increases at lower pH because of decreased charge on impermeant intracellular anions (30–32). The red cell volume increase following exposure of blood to CO_2_ originally observed by Nasse (22) is a consequence of higher intracellular Cl^−^ and $\mathrm{HC}O_{3}^{-}$, but the cell volume change in the physiological $\mathrm{PC}O_{2}$ range is small (30).

**Figure 1.**
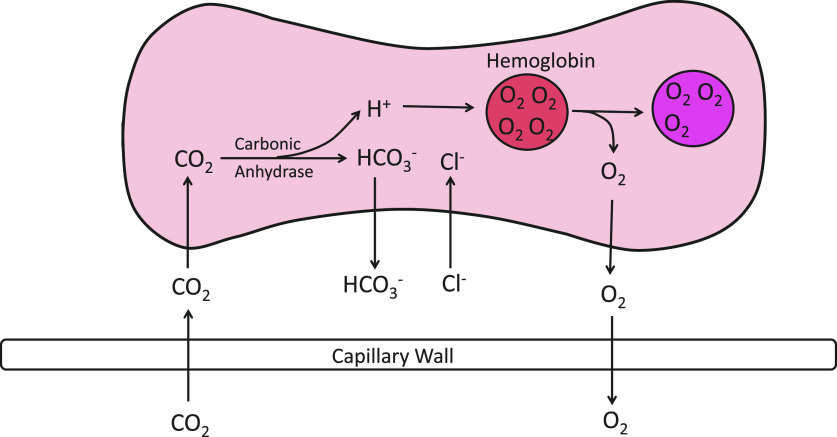
Schematic of the events associated with CO_2_ and O_2_ exchange in a systemic capillary as understood after the discovery of carbonic anhydrase but before band 3 was known to be the anion transporter. CO_2_ diffuses into the cell and is converted to $\mathrm{HC}O_{3}^{-}$ + H^+^ by carbonic anhydrase. The resultant increase in $\mathrm{HC}O_{3}^{-}$ concentration ([$\mathrm{HC}O_{3}^{-}$]) drives efflux of $\mathrm{HC}O_{3}^{-}$, with the charge balanced by influx of Cl^−^. The H^+^ generated by incoming CO_2_ is buffered by hemoglobin, and the slight decrease in pH facilitates O_2_ release.

After the discovery of carbonic anhydrase in the early 1930s (33), there was general agreement that the role of red cell Cl^−^ and $\mathrm{HC}O_{3}^{-}$ transport in capillary CO_2_ exchange is as depicted in Fig. 1 (28, 29, 33–36). In systemic capillaries, incoming CO_2_ is converted to $\mathrm{HC}O_{3}^{-}$ mainly inside the red cells, and the resulting increase in intracellular [$\mathrm{HC}O_{3}^{-}$] drives efflux of $\mathrm{HC}O_{3}^{-}$ in exchange for extracellular Cl^−^. The reverse processes take place in pulmonary capillaries. Under physiological conditions, the half-time for equilibration of $\mathrm{HC}O_{3}^{-}$ and Cl^−^ is <0.1 s (37–39). Red cell Cl^−^/$\mathrm{HC}O_{3}^{-}$ exchange is therefore essentially complete during the capillary transit time (40).

### Electrically Silent Cl^−^ Transport; Substrate Saturation, 1967–1973

Prior to the mid-1960s, red cell anion permeability was described by the fixed charge hypothesis (41) and was assumed to be conductive. This assumption was shown to be incorrect by the demonstration (42) that ionophore-mediated net cation movement in red cells is limited by a Cl^−^ conductance that is far lower than that expected from tracer Cl^−^ exchange rates (43). A separate study by Scarpa et al. (44) used ionophores and pH equilibration experiments to show that Cl^−^/$\mathrm{HC}O_{3}^{-}$ exchange exhibits saturation/competition and is much faster than conductive Cl^−^ transport. Quantitatively, the conductive Cl^−^ permeability P_Cl_ of the human red cell membrane is ∼10,000-fold lower than the permeability expected if the tracer Cl^−^ flux were entirely conductive (45). P_Cl_ is still larger than P_Na_ or P_K_, and the red cell membrane potential is near the equilibrium potential for Cl^−^ (46–49).

The very high rate of Cl^−^ and $\mathrm{HC}O_{3}^{-}$ transport at body temperature makes kinetic studies difficult, and most red cell anion transport experiments in the 1960s used $SO_{4}^{2-}$ or $\mathrm{HP}O_{4}^{2-}$ (41, 50). In 1972 Dalmark and Wieth (51) described a method for measuring red cell ^36^Cl^−^/Cl^−^ exchange at 0–10°C by manual filtration. Gunn et al. (52) used this method in the first detailed kinetic study of red cell Cl^−^ exchange. By varying the intracellular and extracellular [Cl^−^] at a fixed concentration of the weak competitor acetate, they showed that the ^36^Cl^−^/Cl^−^ exchange flux is a saturable function of [Cl^−^] and, unlike $SO_{4}^{2-}$ transport (53), is inhibited by acid pH.

### Revolution in Membrane Biology, 1969–1973

At the same time that red cell Cl^−^ transport was shown to consist mainly of saturable obligatory exchange, there were many other advances in membrane biology, in rapid succession:

- The long-suspected lipid bilayer structure (54) was confirmed by physical measurements (55, 56).
- Electron paramagnetic resonance measurements showed that membrane lipids are more fluid than previously believed (57, 58).
- Cell fusion experiments demonstrated that some membrane proteins have lateral mobility (59).
- Freeze fracture electron microscopy (60) of red cells revealed intramembranous particles, of which band 3 is a major component.
- Chemical labeling experiments demonstrated that a major protein (later determined to be band 3) spans the erythrocyte membrane (61).
- A reproducible method for SDS polyacrylamide gel electrophoresis of red cell membrane proteins was developed (62). The third largest Coomassie-stained polypeptide (∼95 kDa) was called band 3.
- Singer and Nicolson (63) proposed the fluid mosaic model of membrane structure, which replaced the Davson–Danielli–Robertson unit membrane model (64).

### Identification of Band 3 as the Cl^−^/$\mathrm{HC}O_{3}^{-}$ Exchanger, 1974–1979

In the mid-1960s, methods were developed for covalent labeling of the red cell membrane (65, 66). One of the amino-reactive labels, 4-acetamido-4′-isothiocyanatostilbene-2,2′-disulfonate (SITS), was shown by Knauf and Rothstein to be an inhibitor of red cell $SO_{4}^{2-}$ transport (67). Cabantchik and Rothstein (68) extended this work to demonstrate that 4,4′-diisothiocyanatostilbene-2,2′-disulfonate (DIDS) is a very potent transport inhibitor and that [^3^H]4,4′-diisothiocyanatodihydrostilbene-2,2′-disulfonate (H_2_DIDS) (central double bond reduced) labels band 3 in proportion to $SO_{4}^{2-}$ transport inhibition. H_2_DIDS and DIDS have different reactivities, but these differences did not change the conclusion that band 3 is labeled in proportion to transport inhibition (69, 70). Other amino-reactive agents also inhibit anion transport in proportion to band 3 labeling (71, 72).

The studies connecting band 3 with anion transport used $SO_{4}^{2-}$ or $\mathrm{HP}O_{4}^{2-}$ because red cell divalent anion transport is much easier to measure than Cl^−^ transport. At the time, it was unclear whether $SO_{4}^{2-}$ transport and Cl^−^/$\mathrm{HC}O_{3}^{-}$ exchange were mediated by the same system, because of the opposite pH dependence of monovalent versus divalent anion transport (50, 52, 53). Gunn (73) proposed a unifying model in which a critical titratable group can be protonated to convert band 3 from a monovalent to a divalent anion transporter. As a graduate student, I read about Gunn’s titratable carrier model and realized that the model predicted that during net exchange of Cl^−^ for $SO_{4}^{2-}$ there should be H^+^ cotransport with $SO_{4}^{2-}$. Decades earlier, Wilbrandt (74) had shown that suspending Cl^−^-containing red cells in a $SO_{4}^{2-}$ medium caused the extracellular pH to drop because of Cl^−^ exchange with $\mathrm{HC}O_{3}^{-}$. I found that if the medium is purged of atmospheric CO_2_, Cl^−^ efflux into a $SO_{4}^{2-}$ medium causes the extracellular pH to rise as H^+^ is cotransported inward very nearly stoichiometrically with $SO_{4}^{2-}$ (75). This result supported the idea the Cl^−^ and $SO_{4}^{2-}$ are transported by the same system.

Further evidence of a common system for red cell monovalent and divalent anion transport was provided by Weith (76), who showed that the fractional inhibition of ^36^Cl^−^/Cl^−^ and [^14^C]$\mathrm{HC}O_{3}^{-}$/$\mathrm{HC}O_{3}^{-}$ exchange in resealed red cell ghosts is directly proportional to the amount of bound DIDS, as was previously shown for $SO_{4}^{2-}$ (69, 70). In addition, a wide variety of reversibly acting inhibitors have the same effect on the equilibrium exchange of both Cl^−^ and $SO_{4}^{2-}$, measured in the same media and at the same temperature (77). By the end of the 1970s there was general agreement that band 3 is the transporter for Cl^−^, $\mathrm{HC}O_{3}^{-}$, $SO_{4}^{2-}$, and many other inorganic and organic anions in red cells.

## ANION TRANSPORT KINETICS

### Alternating Access; Intrinsic Asymmetry; Single-Turnover Experiments

**Figure 2.**
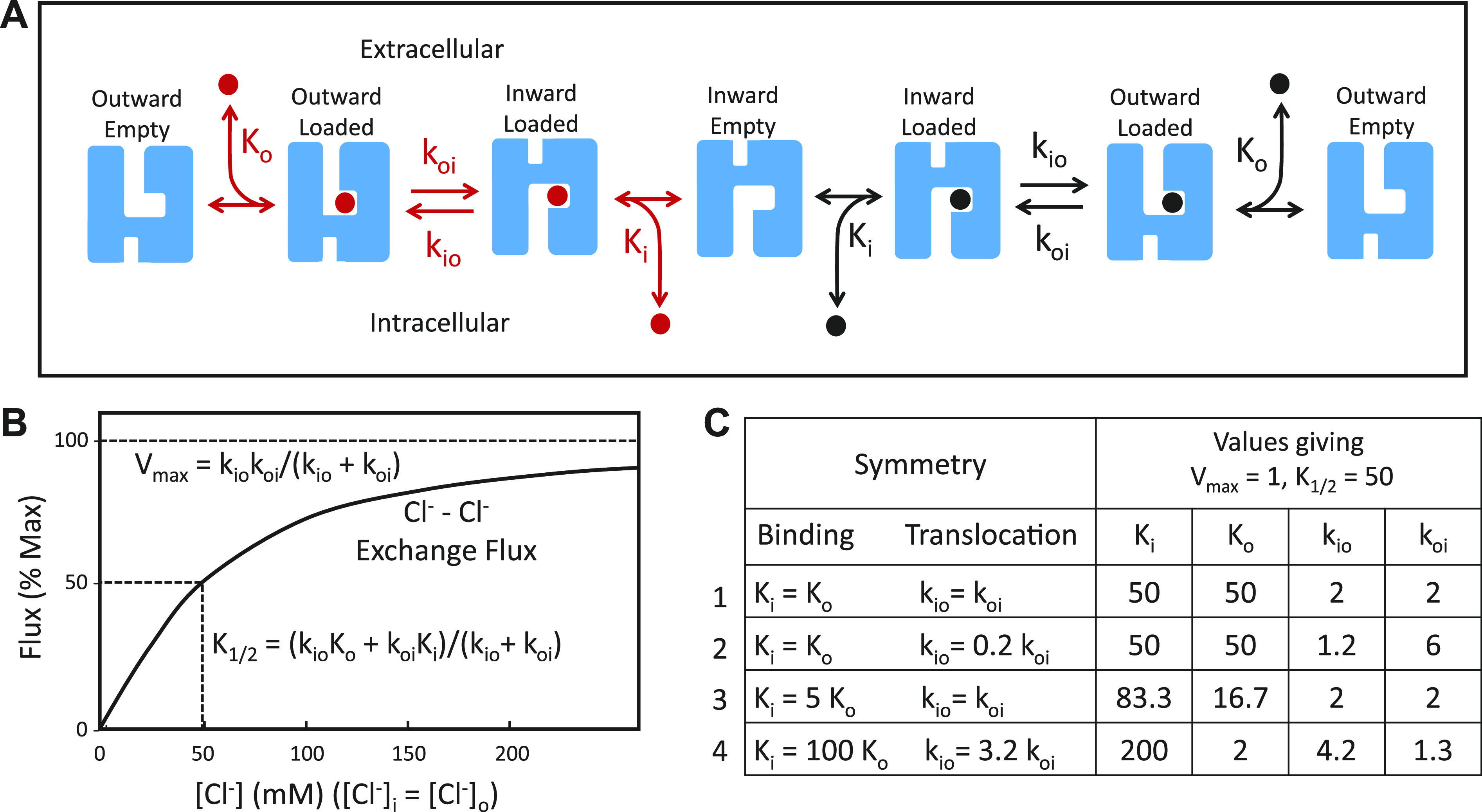
*A*: catalytic cycle for anion exchange by a ping-pong mechanism. The coupling between influx and efflux results from the extremely slow rate of translocation of the empty transporter. The exchange could be between 2 different ions (red and black), with different dissociation and translocation constants, or it could be tracer exchange of the same anion. *B*: ping-pong model prediction for the concentration dependence of Cl^−^/Cl^−^ exchange flux with symmetric Cl^−^ in the absence of competing anions and without any self-inhibition. *C*: 4 combinations of dissociation constants (*K*_i_, *K*_o_) and translocation rate constants (*k*_io_, *k*_oi_) that all result in the same half-maximal concentration (K_1/2_) and *V*_max_ for Cl^−^/Cl^−^ exchange.

Band 3 was an important experimental system for testing the alternating access mechanism of transport (Fig. 2), which had been proposed for pumps by Jardetzky (78). Using anion gradients or transport inhibitors, the population of transporters could be enriched in either outward-facing or inward-facing states, distinguished by binding of chemical probes (79–81). This recruitment of transporters into one or the other state was some of the best early evidence for alternating access in a transporter.

One version of alternating access is a “ping-pong” mechanism (82), in which the outgoing anion is released before binding of the incoming anion (Fig. 2). The ping-pong mechanism was supported by Cl^−^/Cl^−^, Cl^−^/Br^−^, and Br^−^/Br^−^ exchange experiments showing that the substrate concentration (K_1/2_) on one side of the membrane resulting in half-maximal flux depends on the concentration of the exchange partner (83). These experiments as well as studies with inhibitors (84–86) also demonstrated intrinsic asymmetry of the transporter, with more empty transporters facing inward than outward.

The abundance of band 3 (1.2 × 10^6^ polypeptides per cell) makes it possible to prepare resealed red cell ghosts containing only slightly more Cl^−^ ions than band 3 polypeptides. Resuspending these ghosts in medium containing extremely low [Cl^−^] and [$\mathrm{HC}O_{3}^{-}$] causes rapid efflux of ∼0.7 × 10^6^ Cl^−^ ions resulting from a half-turnover of the catalytic cycle (87). This experiment showed that the Cl^−^ efflux event is possible without influx. The number of Cl^−^ ions released is consistent with the idea that a complete catalytic cycle is one pair of anions exchanging per copy of band 3 polypeptide. Further evidence for a ping-pong mechanism is the transient uphill ^36^Cl^−^ efflux induced by adding H_2_DIDS to red cells containing very low [Cl^−^] (88).

### Self-Inhibition

Although there is a large body of evidence for a ping-pong mechanism, the kinetics of band 3-mediated transport are complex. The exchange flux of Cl^−^ and other halides decreases at high concentrations (83, 89, 90), implying that there is an anion binding event that inhibits at least one step in the alternating access catalytic cycle. The self-inhibition site has a higher affinity for I^−^ than for Cl^−^ (89), and the site is inactivated by deprotonation of a group with pK ∼11 at 0°C (90). Self-inhibition can be modeled as a noncompetitive inhibitory site on each band 3 subunit, but as Salhany and coworkers pointed out, other mechanisms are possible, including those with a ternary complex involving both exchanging anions (91) and site-site interactions resulting in negative cooperativity (92).

### Estimates of Cl^−^ and $\mathrm{HC}O_{3}^{-}$ Affinities

With symmetric Cl^−^ and $\mathrm{HC}O_{3}^{-}$ concentrations totaling 165 mM, the Cl^−^/Cl^−^ exchange flux is a concave upward function of [Cl^−^] and the $\mathrm{HC}O_{3}^{-}$/$\mathrm{HC}O_{3}^{-}$ exchange flux is a concave downward function of [$\mathrm{HC}O_{3}^{-}$], indicating that the band 3 $\mathrm{HC}O_{3}^{-}$ affinity is approximately fourfold higher than that of Cl^−^ (76). Self-exchange experiments with symmetric Cl^−^ or $\mathrm{HC}O_{3}^{-}$ as the only permeant anion are also consistent with $\mathrm{HC}O_{3}^{-}$ affinity being higher than that of Cl^−^ (76, 93).

It is inherently difficult to estimate absolute Cl^−^ and $\mathrm{HC}O_{3}^{-}$ affinities from transport measurements. In a self-exchange with symmetric [Cl^−^], the same measured *V*_max_ and K_1/2_ can result from an infinite number of combinations of true dissociation constants (*K*_i_ and *K*_o_) and translocation rate constants (*k*_io_ and *k*_oi_); some examples are in Fig. 2. The same thing is true for exchange experiments with Cl^−^ varied on only one side of the membrane; K_1/2_ depends on both binding affinity and translocation rate constants. Gunn, Fröhlich, Knauf, and coworkers used a combination of specific transport inhibitors and detailed kinetic analysis to provide strong evidence that Cl^−^ binding to band 3 transport sites is fairly symmetric (*K*_i_ ≅ *K*_o_), but *k*_oi_ (inward translocation) is larger than *k*_io_ (83, 85, 94), similar to *line 2* of the table in Fig. 2*C*. In contrast to Cl^−^, binding of $\mathrm{HC}O_{3}^{-}$ appears to be asymmetric (*K*_o_ << *K*_i_), and *k*_oi_ is smaller than *k*_io_ (95), similar to *line 4* of the table in Fig. 2*C*. Despite the asymmetries in substrate binding and/or translocation, with the relatively small gradients of Cl^−^ and $\mathrm{HC}O_{3}^{-}$ found in pulmonary and systemic capillaries, the time course of net exchange is predicted to be very similar in each direction (95).

Because of the abundance of band 3, it is possible to use NMR to measure Cl^−^ binding (96–100) and to distinguish among different classes of transport inhibitors (101–103). The NMR results are consistent with a ping-pong model in which translocation rather than binding/release is rate limiting (104), Cl^−^ binding is symmetric (K ∼ 50 mM), and inward Cl^−^ translocation is faster than outward (100). NMR measurements of $NO_{3}^{-}$ binding indicate that the dissociation constants for $NO_{3}^{-}$ and $\mathrm{HC}O_{3}^{-}$ binding to transport sites on band 3 are 5–10 mM (105), in agreement with transport measurements showing a higher affinity for $\mathrm{HC}O_{3}^{-}$ than for Cl^−^ (76, 93). With an approximately four times higher $\mathrm{HC}O_{3}^{-}$ affinity, the fractional occupancy of transport sites with $\mathrm{HC}O_{3}^{-}$ and Cl^−^ should be similar in vivo, because the Cl^−^ concentration is approximately four times higher.

### Affinities and Translocation Rates of Other Band 3 Substrates

Table 1 lists many of the known substrates of mammalian band 3, in very rough order of transport rates. For divalent or pH-titratable anions, the rates are at the pH optimum for transport of the ion (106). There are very large (>1,000-fold) variations in transport rates among different anions, with those similar to Cl^−^ or $\mathrm{HC}O_{3}^{-}$ (e.g., Br^−^, formate, $NO_{3}^{-}$) transported most rapidly. Oxyanions with tetrahedral geometry are transported more slowly than planar trigonal anions (107–109).

**Table 1.** Approximate band 3-mediated X^−^/X^−^ or Cl^−^/X^−^ exchange rates relative to Cl^−^/Cl^−^ exchange

Relative Rate	Anion (References)	Additional Information
1	Cl^−^, $\mathrm{HC}O_{3}^{-}$ (76, 224, 352)	Transported at similar rates; $\mathrm{HC}O_{3}^{-}$ has higher affinity for transport site.
0.3–0.7	$NO_{3}^{-}$ (493)	Fast, similar to $\mathrm{HC}O_{3}^{-}$.
	Formate (HCOO^−^) (108, 225, 227)	Slightly slower than $\mathrm{HC}O_{3}^{-}$. Also transported as free acid.
	$NO_{2}^{-}$ (494, 495, 513, 514)	Rate not known exactly because of parallel HNO_2_ transport. Could be similar to formate.
	Br^−^ (83)	Cl^−^/Br^−^ exchange is faster than Br^−^/Br^−^ exchange, as predicted by ping-pong mechanism.
	HS^−^ (515)	Measured as Jacobs–Stewart cycle of rapid transport of both HS^−^ and H_2_S.
0.1–0.3	Oxalate (^−^OOCCOO^−^) (227, 516, 517)	Fastest divalent anion transported by band 3.
	Superoxide ($O_{2}^{-}$) (518)	Transport rate not clear but probably fast.
	Peroxynitrite (OONO^−^) (375, 496, 497)	Causes oxidative damage of band 3 and reduced transport. Undissociated acid also transported.
	F^−^ (43)	Slower than Br^−^.
	OH^−^ (90)	Detectable but hard to quantify because very high pH inhibits monovalent anion transport.
	Selenite ($\mathrm{HSe}O_{3}^{-}$) (107, 519)	Possible connection with arsenite toxicity.
0.03–0.1	Malonate (^−^OOCCH_2_COO^−^) (516, 520)	Almost as fast as oxalate; larger dicarboxylates are slower.
	I^−^ (493)	Slow, but has high affinity for self-inhibitory site, so some of slow rate could be self-inhibition.
	Thiocyanate (SCN^−^) (51)	Inhibits Cl^−^ transport strongly.
	Bisulfite ($\mathrm{HS}O_{3}^{-}$) (521)	Much faster than $SO_{4}^{2-}$.
	Phosphite ($H_{2}PO_{3}^{2-}$) (106, 109)	Much faster than $H_{2}PO_{4}^{-}$.
	Borohydride ($BH_{4}^{-}$) (522)	Enters cells in <1 min at 3°C, but rate not quantified.
0.01–0.03	Hypophosphite ($PO_{2}^{-}$) (106, 109)	Slower than $PO_{3}^{2-}$.
	Glyoxylate (HCOCOO^−^) (227)	Much slower than $\mathrm{HC}O_{3}^{-}$.
	Glycolate (HOCH_2_COO^−^) (227)	Much slower than $\mathrm{HC}O_{3}^{-}$.
	Fluorophosphate ($\mathrm{FP}O_{3}^{-}$) (109)	Slower than planar oxyanions of phosphorus.
	Acetate (H_3_CCOO^−^) (225, 493)	Hard to quantify because of rapid free acid transport. Used as spectator anion.
0.003–0.01	Selenate ($\mathrm{Se}O_{4}^{2-}$) (107, 519)	Much slower than selenite.
	Vanadate (523)	Rate not known precisely; inhibits ATPases and PTPs.
0.001–0.003	Dithionite ($S_{2}O_{4}^{2-}$) (91)	Measured as exchange with $SO_{4}^{2-}$.
	Pyruvate (225, 524)	Also transported by monocarboxylate transporter.
	Sulfate ($SO_{4}^{2-}$) (118)	Measured at very low extracellular pH; much slower at neutral pH.
<0.001	Chromate ($\mathrm{Cr}O_{4}^{2-}$) (525, 526)	Influx facilitates labeling red cells with ^51^Cr for red cell lifetime measurements.
	Glycine anion (H_2_NCH_2_COO^−^) (527)	Slower than glycolate.
	$H_{2}PO_{4}^{-}$/$\mathrm{HP}O_{4}^{2-}$ (50, 106, 109, 528, 529)	Also transported by Na^+^-coupled cotransporter.
	Phosphoenolpyruvate (240, 530)	Only known glycolytic intermediate transported across red cell membrane.
	Lithium carbonate ($\mathrm{LiC}O_{3}^{-}$) (531, 532)	Under physiological conditions represents over half the lithium flux in red cells.
	Pyridoxal phosphate (241)	Also reacts with K851.
	NBD-taurine (242)	Used to measure transport by fluorescence
	Taurine monochloramine (533)	Produced from taurine by neutrophil myeloperoxidase.

NBD-taurine, 2-[*N*-(7-nitrobenz-2-oxa-1,3-diazol-4-yl)amino] ethanesulfonate; PTP, protein tyrosine phosphatase.

For anions that are transported much more slowly than Cl^−^, it is possible to estimate binding affinity for outward-facing transport sites by measuring the initial Cl^−^ efflux into media containing only the slowly transported anion X^−^. The [X^−^]_o_ that gives half-maximal flux provides an estimate of the dissociation constant of X^−^ for outward-facing sites, because the catalytic cycle is limited by X^−^ influx rather than by Cl^−^ efflux (87). The range of binding affinities determined from Cl^−^/X^−^ exchange (∼10-fold) is much more limited than the >1,000-fold variations in maximum transport rates (107). The large variations in transport rate are likely a consequence of how each substrate interacts with the transition state between inward-facing and outward-facing states (107, 110, 111).

### Is OH^−^ a Substrate for Band 3?

In 1932 Jacobs and Parpart (112) estimated the rates of entry of acid into red cells and concluded, based on a mass action argument, that the data are much more consistent with OH^−^ efflux than with H^+^ influx. Given the high rates of transport of other monovalent anions, it would not be surprising if OH^−^ is a substrate of band 3. However, as shown by Jacobs and Stewart (113), if small amounts of CO_2_ are present, a repeated cycle of $\mathrm{HC}O_{3}^{-}$ efflux, extracellular dehydration, CO_2_ influx, and intracellular hydration will result in net net efflux of OH^−^ equivalents. Therefore, what appears to be Cl^−^/OH^−^ exchange could actually be the result of atmospheric CO_2_ acting through the Jacobs–Stewart cycle. Wieth and Bjerrum (90) measured a relatively slow OH^−^ influx in red cells at extracellular pH 12.4, which does not cause irreversible damage to band 3 at 0°C. At this pH the Jacobs–Stewart cycle should not operate because extracellular CO_2_ is almost entirely in the form of $CO_{3}^{2-}$. Therefore, the base influx at very high extracellular pH is likely actual OH^−^ transport by band 3, but it is difficult to make a quantitative comparison between OH^−^ and Cl^−^ fluxes, because raising the extracellular pH to produce an appreciable [OH^−^] also inhibits monovalent anion exchange (90).

### Additional Transport Modes

Although the main transport process carried out by band 3 is 1:1 exchange of monovalent anions, there are other, slower, modes of transport:

- Anion conductance. Band 3 mediates most of the red cell conductive Cl^−^ flux (114), which is not a result of reorientation of the empty transporter (115, 116). Band 3 also catalyzes conductive transport of $SO_{4}^{2-}$ and of OH^−^ (114).
- H^+^-$SO_{4}^{2-}$ cotransport. As predicted by the titratable carrier model (above), H^+^ is cotransported with $SO_{4}^{2-}$ during Cl^−^/$SO_{4}^{2-}$ exchange (75). H^+^ and $SO_{4}^{2-}$ can bind to the transporter in either order, with the first bound increasing the affinity for the second by ∼ 10-fold (117, 118).
- H^+^-Cl^−^ cotransport. In the pH range 5.7–7.4 in media purged of atmospheric CO_2_, red cell pH equilibration rates are much more consistent with H^+^-Cl^−^ cotransport than with Cl^−^/OH^−^ exchange; the apparent H^+^-Cl^−^ cotransport is much slower than Cl^−^/Cl^−^ exchange but is inhibited by low concentrations of DIDS (119). Kinetic studies with chemically modified band 3 suggest that H^+^-Cl^−^ cotransport results from a slow event in which 2 Cl^−^ ions are translocated on the protonated (divalent) form of band 3 (120).
- K^+^ transport. At low ionic strength, red cells exhibit a stilbenedisulfonate-sensitive monovalent cation permeability that is very likely mediated by band 3 (121). As discussed below, some variant forms of band 3 can transport cations.
- Stilbenedisulfonate transport. At very low pH, band 3 can transport stilbenedisulfonates, which are normally impermeant (122).

## BAND 3 STRUCTURE

### cDNA Sequence, Expression Pattern, Related Proteins, Nomenclature

Proteolytic fragments of band 3 were sequenced by Edman degradation in the early 1980s (123–125), but it would have taken many more years to sequence the entire protein. Fortunately, cDNA technology was advancing rapidly, and one of the first transport protein cDNAs to be sequenced was mouse band 3 (126). The human band 3 cDNA sequence (127, 128) and the mouse and human gene sequences (129–131) were published shortly afterward.

The main sites of mammalian band 3 gene expression are erythroid cells and the kidney, but it is also expressed in the heart (132), and there is recent evidence that band 3 is involved in sperm capacitation (133). In the kidney, the band 3 gene is transcribed with an alternate promoter (134, 135) resulting in an NH_2_ terminus that is 65 residues shorter than red cell band 3 (136). Renal band 3 mediates Cl^−^/$\mathrm{HC}O_{3}^{-}$ exchange, resulting in $\mathrm{HC}O_{3}^{-}$ efflux across the basolateral membrane of acid-secreting α intercalated cells in cortical and medullary collecting tubules (137, 138).

Soon after band 3 cDNA was sequenced, related transcripts and proteins were identified. To clarify the terminology, Ron Kopito, at a 1989 meeting hosted by Naotaka Hamasaki, proposed the name AE1 (Anion Exchanger 1) for band 3, AE2 for the widely expressed homolog (139–141), and AE3 for the neural homolog (142). AE2 and AE3 are, like AE1, Na^+^-independent Cl^−^/$\mathrm{HC}O_{3}^{-}$ exchangers. The terms “AE1” and “band 3” are used interchangeably; “AE1” is used more often in heterologous expression studies, comparisons among Cl^−^/$\mathrm{HC}O_{3}^{-}$ exchangers, and work on the product of the renal transcript kAE1. AE1 is also called capnophorin (143) for its role in $\mathrm{HC}O_{3}^{-}$ transport. Fifty years after Fairbanks et al. (62), the term “band 3” is still commonly used for the red cell protein.

### Posttranslational Modifications

Band 3 has one site of *N*-glycosylation (144) at N642, located in a long extracellular loop between the 7th and 8th transmembrane helices. The carbohydrate is heterogeneous, causing band 3 to run as a broad zone on SDS-PAGE (145–148). Enzymatic deglycosylation does not inhibit anion exchange (149). Band 3 is fatty acylated at C843 (150). Acylation of this residue is not required for trafficking of AE1 to the cell surface (151) or for anion transport (152). Phosphorylation sites are discussed below.

### Cytoplasmic and Membrane Domains

**Figure 3.**
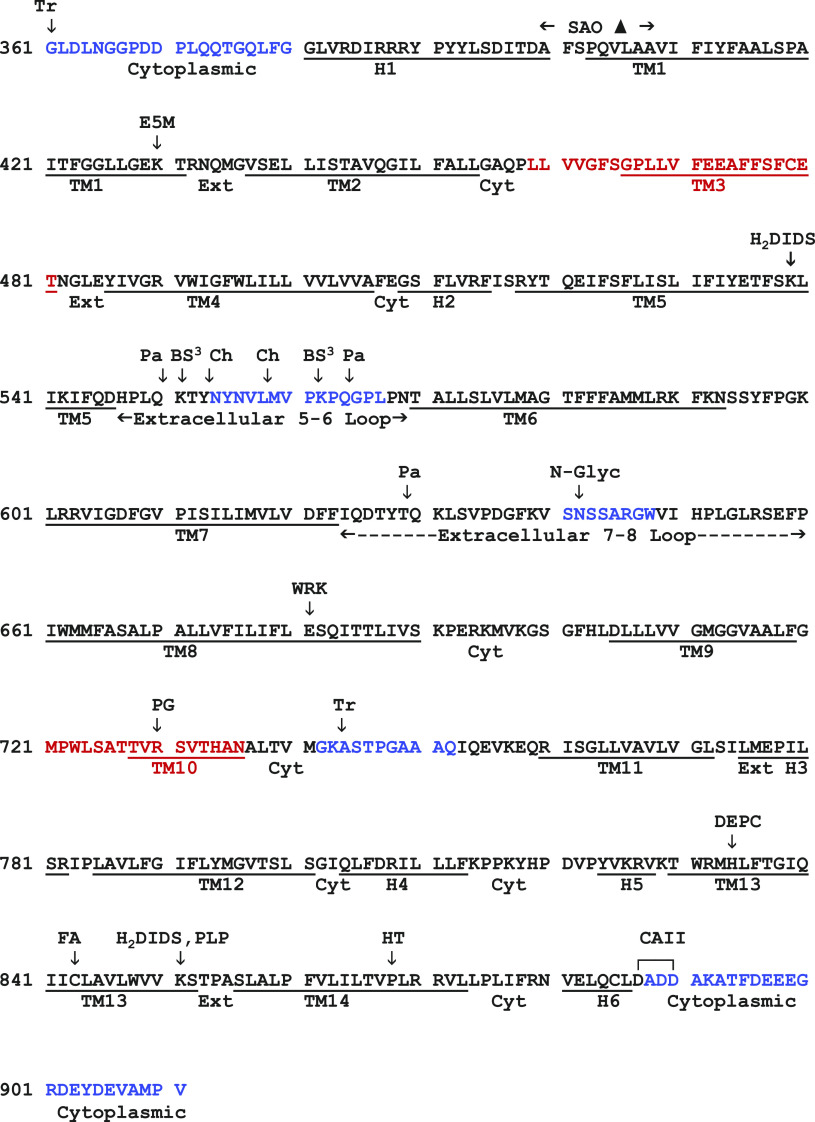
Amino acid sequence of the membrane domain of band 3. Sites of *N*-glycosylation (N-Glyc) (N642) and fatty acylation (FA) (C843) are indicated. Underlined sequences are membrane α-helices (TM1–TM14) and surface α-helices (H1–H6). Sequences that are not ordered in the crystal structure are shown in blue. The 2 transmembrane sequences (TM3 and TM10) that include both helical and nonhelical segments are in red. Arrows indicate sites of proteolytic cleavage by trypsin (Tr), chymotrypsin (Ch), and papain (Pa); chemical modification by eosin-5-maleimide (E5M), 4,4′-diisothiocyanatodihydrostilbene-2,2′-disulfonate (H_2_DIDS), bis(sulfosuccinimidyl)suberate (BS^3^), diethylpyrocarbonate (DEPC), Woodward’s reagent K (WRK), phenylglyoxal (PG), pyridoxal phosphate (PLP), and band 3 HT point mutation. Locations of the Southeast Asian ovalocytosis (SAO) deletion and DADD sequence binding carbonic anhydrase II (CAII) are also indicated. References are in text.

Human band 3 consists of a water-soluble NH_2_-terminal cytoplasmic domain (360 residues) and a mainly hydrophobic COOH-terminal domain (551 residues) with a short COOH-terminal hydrophilic sequence (127, 128). The cytoplasmic domain is an attachment site for the membrane skeleton via ankyrin (153) and binds hemoglobin and glycolytic enzymes (10). The membrane domain carries out anion exchange; its amino acid sequence is in Fig. 3, annotated with locations of various markers.

### Dimeric Structure

Although band 3 can be monomeric (154–158), it is most likely dimeric in normal intact membranes, with some dimers associating into tetramers. The first evidence for dimers was that Cu^2+^/*o*-phenanthroline (CuP) cross-links band 3 to an –S–S– dimer (159). The cross-linked cysteine residues are in the cytoplasmic domain (160–162). The purified cytoplasmic domain is dimeric without cross-linking (163). Crystal (164, 165) and solution (166) structures of the cytoplasmic domain show a tightly associated dimer. The sulfur atoms of the only two cysteines (C201 and C317) are ∼20 Å from each other in adjacent subunits of the crystal structure (164, 165). The cytoplasmic domain undergoes a pH-dependent conformational change (167, 168), which could move the side chains close enough to each other for –S–S– cross-linking.

**Figure 4.**
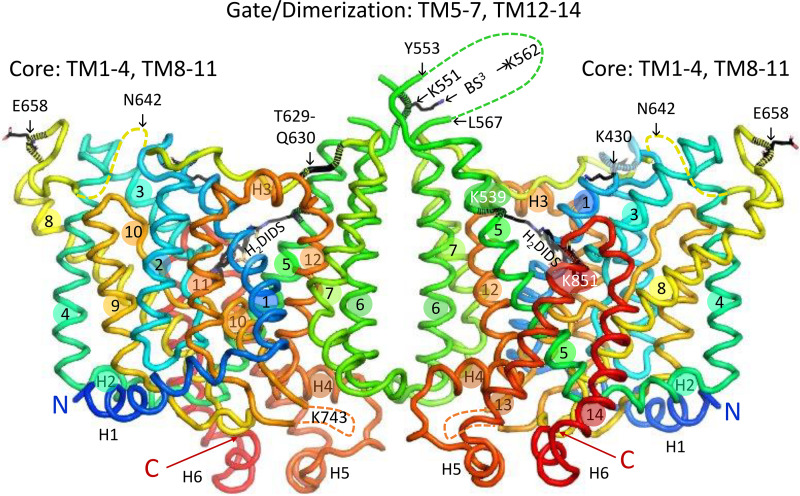
Ribbon representation (PyMOL, pdb 4YZF) of the crystal structure of the membrane domain dimer (18), viewed in the plane of the membrane. Membrane α-helices TM1–TM14 are labeled with numbers in colored circles, and surface helices are labeled H1, etc. The NH_2_-terminal end of H1 preceded by an unresolved sequence is indicated, as is the COOH-terminal end of H6, which is followed by the unresolved COOH-terminal 24 residues. The 3 internal unresolved sequences are shown as dashed curves. The locations of several extracellular biochemical markers and proteolysis sites are indicated. The glycosylation site N642 is in the unresolved sequence between TM7 and TM8. The helices connecting core and gate are H2 on the cytoplasmic side (foreground on the right-hand subunit) and H3 on the extracellular side (foreground on the left-hand subunit). Residues in the membrane domain that can be cross-linked to the cytoplasmic domain are all in the gate/dimerization domain except for K743 in the disordered sequence between TM10 and TM11, which can be cross-linked to 2 different residues in the cytoplasmic domain (187).

**Figure 5.**
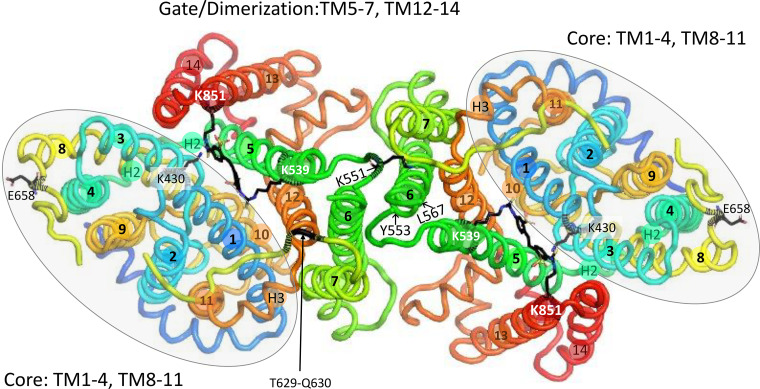
Ribbon representation of the band 3 dimer crystal structure, viewed from the extracellular side of the membrane to illustrate the relationships among dimer interface, gate, core, and stilbenedisulfonate site. The core domains are in the shaded ovals. Covalently bound 4,4′-diisothiocyanatodihydrostilbene-2,2′-disulfonate (H_2_DIDS) between core and gate is shown in black sticks for both subunits. One of the connections between core and gate domains is cytoplasmic surface helix H2, which is largely obscured by TM8 in this view; the two ends of H3 are labeled. The second connection between core and gate consists of extracellular helix H3 and the extracellular TM7-TM8 loop. Papain cleavage of the TM7-TM8 loop between T629 and Q630 (shown for the left-hand subunit) appears to stabilize the inward-facing conformation (see text).

The isolated membrane domain has high α-helix content (169) and is dimeric (170–173). In the crystal structure of the dimer (18), each subunit has 14 membrane-spanning (TM) segments, with subdomains designated core and gate, the terms originally chosen to describe the structurally related bacterial uracil transporter UraA (174). Figure 4 is a ribbon representation (PyMOL) of the membrane domain dimer, viewed from within the membrane. Figure 5 is the same structure viewed from the extracellular side.

The core domain consists of TM1–TM4 and TM8–TM11 arranged in an inverted repeat. TM2, -4,-9, and -11 are nearly perpendicular to the membrane on the side of the core facing away from the gate. TM3 and TM10 both contain helices that go partway through the membrane. The NH_2_-terminal ends of these helices face each other roughly in the middle of the membrane and are separated by a gap that is the likely substrate anion binding site (see below). TM1 and TM8 are relatively long and are located on each side of the TM3/TM10 helix. The gate domain consists of TM5–TM7 and TM12–TM14, also in an inverted repeat. The gate is connected to the core via cytoplasmic helix H2 between TM4 and TM5 and extracellular helix H3 between TM11 and TM12. The other connection between core and gate is the long extracellular sequence between TM7 and TM8.

The gate domain contains the dimer interface. The TM5-TM6 loops of both subunits are in close proximity to each other, consistent with cross-linking experiments indicating that bis(sulfosuccinimidyl)suberate (BS^3^), a membrane-impermeant cross-linker (175, 176), forms an intermolecular cross-link involving K551 and/or K562 (177). At the intracellular surface, the TM6-TM7 loop and COOH terminus of H4 of opposite subunits are adjacent to each other. In the membrane interior, there is a cavity between the subunits that molecular dynamics simulations suggest is occupied by lipid, especially cholesterol (178).

### Interaction between Cytoplasmic and Membrane Domains

A large body of evidence indicates that the band 3 cytoplasmic domain is connected to the membrane domain by a flexible tether, without stable noncovalent associations between domains (179, 180):

- The cytoplasmic domain is released from the membrane by proteolysis under mild conditions (144, 181, 182).
- Calorimetry demonstrates independent thermal unfolding of the two domains (183).
- Proteolytic removal of the cytoplasmic domain does not have a major effect on transport (184, 185).
- The membrane domain, when expressed in either *Xenopus* oocytes (186) or HEK 293 cells (165), mediates Cl^−^ exchange that is indistinguishable from that of the whole protein.
- Mutations that affect the conformation of the cytoplasmic domain do not affect expression of transport activity in HEK cells (165).

However, recent findings have shown that interactions between membrane and cytoplasmic domains are more extensive than previously believed. Rivera-Santiago et al. (187) demonstrated that several zero-length cross-links can be formed between the membrane and cytoplasmic domains of human red cell band 3. The cross-links mainly involve the gate/dimerization region of the membrane domain, and in a structural model of the intact protein the cytoplasmic domain does not appear to interfere with cytoplasmic access of substrate anions (187). The only cross-linked core residue is K743, which can be cross-linked to two different carboxylate side chains in the cytoplasmic domain (187). This residue is in a sequence that is not ordered in the crystal structure but is close to the gate domain (Fig. 4). The fact that some of the cross-links involve two or more partners is evidence that the interactions between the two domains are dynamic. Functional interaction between the two domains is discussed below in reference to tyrosine phosphorylation.

### In Situ Proteolysis

**Figure 6.**
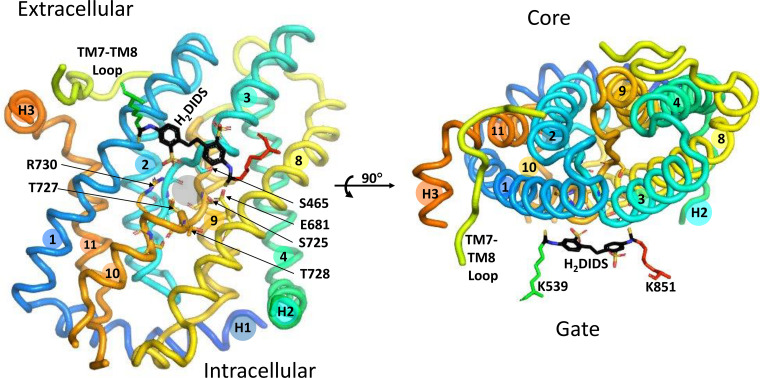
*Left*: core domain, viewed from the gate/dimerization domain, showing the relationship between the probable substrate binding pocket (gray circle between the helical portions of TM3 and TM10) and covalently bound 4,4′-diisothiocyanatodihydrostilbene-2,2′-disulfonate (H_2_DIDS) (black sticks). The side chains of R730 and E681 are on either side of the substrate pocket. One of the H_2_DIDS sulfonate groups is near the side chain of R730. The positions of other polar side chains (S465, S725, T727, and T728) are indicated. The 3 links between core and gate domains (portion of TM7-TM8 loop and surface helices H2 and H3) are viewed end-on. *Right*: same structure, rotated to show the view from the extracellular medium. The only gate domain amino acid residues shown are those covalently bound to H_2_DIDS. The helical portion of TM10 is behind TM1 in this view. H_2_DIDS is between core and gate, near but not in the substrate binding pocket.

Band 3 has been the subject of numerous in situ proteolysis studies. Most of the known proteolysis sites of native band 3 are in sequences that are disordered in the crystal structure (Figs. 3 and 4):

- Connection between cytoplasmic and membrane domain: The membrane domain NH_2_ terminus is G361 (123), and the next 19 residues leading to helix H1 are disordered in the crystal.
- TM5-TM6 exofacial loop: Extracellular chymotrypsin or thermolysin produces an NH_2_-terminal ∼60-kDa fragment with a COOH-terminal tyrosine residue, now known to be Y553 (181, 188, 189), adjacent to a disordered sequence in the crystal structure (dashed green loop in Fig. 4). Proteolysis of the TM5-TM6 loop does not inhibit anion transport (190, 191) or stilbenedisulfonate binding (192). Expression of fragments confirmed that transport does not require this loop to be intact (193).
- TM7-TM8 exofacial loop: The sequence (I624–P660) between TM7 and TM8 (yellow in Fig. 4, Fig. 5, and Fig. 6) is ordered in the crystal structure except for a short disordered segment (dashed yellow in Fig. 4) containing the *N*-glycosylation site. Digestion of cells with papain (194) or isolated membranes with pepsin (124) cleaves between T629 and Q630. This is the only in situ proteolysis site of native band 3 that is resolved in the crystal structure; it is located close to the H3 connection between TM11 (core) and TM12 (gate). Papain cleavage of this site inhibits transport if measured with symmetric anion concentrations (191, 195) but accelerates transport measured under influx-limiting conditions (196). This could be explained if papain cleavage at T629–Q630 destabilizes the outward-facing state.
- TM10-TM11 cytoplasmic loop: Trypsin, at low ionic strength, cleaves the cytoplasmic surface of band 3 at K743 (197, 198). Glycosylation scanning in a cell-free translation system (199) indicated that this loop is extracellular, but the loop is not glycosylated when expressed in the plasma membrane of HEK 293 cells (200), suggesting that the TM10-TM11 loop may be transiently exposed to the endoplasmic reticulum (ER) lumen during synthesis/insertion of band 3 (20).
- COOH-terminal hydrophilic region: Carboxypeptidase Y digestion experiments demonstrated that the COOH terminus is cytoplasmic (201).
- Sites exposed by low or high pH: In situ proteolysis of band 3 exposed to high or low pH has provided information about the stability of transmembrane helices. Pepsin removes most of the surface loops and produces stable membrane-bound fragments (124, 202). Hamasaki and coworkers used in situ proteolysis following exposure of intact membranes to 10–100 mM NaOH to identify band 3 sequences that are more likely to be surrounded by protein than lipid (203–206).

### Stilbenedisulfonate Binding Site; Intramolecular Cross-Link

The stilbenedisulfonate binding site is located between the core and gate of each subunit of the dimer (Fig. 5). Prior to covalent reaction, DIDS and H_2_DIDS bind reversibly with high affinity (69, 70, 207). Many other stilbenedisulfonate derivatives can bind to this site (208–215). The site is accessible only from the extracellular medium and is in a cleft below the membrane surface, consistent with the most recent fluorescence resonance energy transfer (FRET) data (216). The main H_2_DIDS covalent attachment site is a reactive amino group in the 60-kDa chymotryptic fragment (residues 1–553) (190, 217). This residue (human K539; mouse K558) is not necessary for anion exchange (218–220).

As a postdoctoral fellow with Hermann Passow, I consistently found that if cells were treated with H_2_DIDS some of band 3 appears to be resistant to chymotrypsin cleavage into the 60-kDa and 35-kDa fragments. It seemed possible that this apparent resistance was the result of H_2_DIDS, a bifunctional reagent, forming a covalent intramolecular cross-link between the two fragments. To test this idea, I incubated H_2_DIDS-treated cells at high extracellular pH (9.5) and found that essentially all copies of band 3 migrated at 95 kDa on gels, even if it had previously been cleaved by extracellular chymotrypsin (191).

This result showed that, after covalent attachment of H_2_DIDS to the 60-kDa fragment, the other –N=C=S group can react with the 35-kDa fragment at high pH to produce an intramolecular cross-link. It also showed that there is one H_2_DIDS binding site per band 3 polypeptide. Several years later I collaborated (my role was minor) with the group led by Naotaka Hamasaki to identify the site of H_2_DIDS cross-linking as K851 (221), which is also the site of covalent modification by pyridoxal 5′-phosphate (222).

The H_2_DIDS cross-linking reaction proved to be a useful tool for studying band 3. The membrane domain crystals (18) were prepared from band 3 that had been internally cross-linked by H_2_DIDS at high pH. It is known that very high extracellular pH inhibits red cell Cl^−^/Cl^−^ exchange (90, 223, 224), but even at pH 10 Cl^−^/Cl^−^ exchange is inhibited only 50% relative to neutral pH. It is of course possible that the H_2_DIDS-cross-linked structure does not represent a native conformation, but it is more likely to be similar though perhaps not identical to the outward-facing native conformation.

### Evidence for Independent Transport by Dimer Subunits

There is a very well-established linear relationship between bound DIDS or H_2_DIDS and inhibition of red cell anion transport (70, 76, 225–230). Covalent reaction of a portion of band 3 by stilbenedisulfonate lowers the *V*_max_ but does not change the K_1/2_ for transport (231). The simplest interpretation of these findings is that, in the band 3 dimer, one subunit can transport normally even if the other subunit is completely inhibited by DIDS. It is also possible that that stilbenedisulfonate binding disrupts preexisting subunit interactions (232).

Independent transport by the two subunits of the dimer is consistent with the crystal structure. H_2_DIDS is bound covalently to residues in the gate domain, but the binding site is not near the dimer interface (Fig. 5). Cross-linking the TM5-TM6 loops of adjacent subunits with BS^3^ does not inhibit anion transport (233), suggesting that the subunit interface is not closely involved in anion binding or translocation. The subunit interface may represent a relatively fixed scaffold, with the transport-related conformational changes resulting from each core domain moving independently. Additional evidence that the subunit interface is not necessary for transport is that expression of a construct lacking TM6 and TM7 mediates both anion exchange and conductance (234).

### Substrate Binding Site

Stilbenedisulfonates bind reversibly with high affinity only to the outward-facing conformation of band 3 (87, 235), and binding is competitive with Cl^−^, as measured by transport (211, 236) and NMR (97). However, the stilbenedisulfonate site and the Cl^−^ binding site are not identical; Cl^−^ interferes with 4,4′-dibenzamidostilbene-2,2′-disulfonate (DBDS) binding by increasing the DBDS dissociation rate rather than by occupying the same site (237, 238).

The membrane domain crystal structure has no bound substrate anion, but the substrate binding site is likely located between the NH_2_-terminal ends of the α-helical portions of TM3 and TM10 (18, 20), by analogy with UraA (174). This location is close to but distinct from the site of covalently bound H_2_DIDS (Fig. 6). The substrate binding pocket is roughly halfway through the membrane, consistent with evidence that the substrate traverses ∼10–15% of the transmembrane electrical field in moving from the extracellular medium to the binding site (239).

As discussed above, variations in binding affinity among different substrates are smaller than the variations in translocation rates. Anions as large as phosphoenolpyruvate (240), pyridoxal phosphate (241), and *N*-(2-aminoethylsulfonate)-7-nitrobenz-2-oxa-3-diazole (NBD)-taurine (242) are band 3 substrates. Modeling studies will be necessary to gain insight into how anions of various sizes and geometries fit into the binding pocket.

### Site of Eosin-5-Maleimide Labeling

Eosin-5-maleimide (E5M) is used clinically to estimate the amount of band 3 per cell (243) and has been used extensively to measure band 3 rotational mobility (see below). E5M reacts covalently with K430 (244, 245) on the extracellular end of TM1 near the DIDS binding pocket but closer to the extracellular surface (Fig. 4 and Fig. 5). The E5M covalent reaction is preceded by reversible binding to a site in the outward-facing conformation that is distinct from the transport site (246, 247).

### Role of E681 and R730

The pH dependence of $SO_{4}^{2-}$ influx during Cl^−^/$SO_{4}^{2-}$ exchange (118) suggested that a carboxyl group on band 3 binds the H^+^ that is cotransported with $SO_{4}^{2-}$. Matt Anderson and I used the arylsulfonic acid Woodward’s reagent K (WRK) to try to identify the carboxyl group. WRK was known to form an enol ester adduct with protein carboxyl groups (248), and we showed that that [^3^H]$BH_{4}^{-}$ can reductively cleave the enol ester to produce a radiolabeled alcohol in place of the original carboxylate (249).

The functional effects of this modification (inhibition of Cl^−^ transport, stimulation of electrogenic Cl^−^/$SO_{4}^{2-}$ exchange) were consistent with the idea that WRK/$BH_{4}^{-}$ converts the glutamate residue associated with H^+^-$SO_{4}^{2-}$ cotransport into an alcohol (250, 251). The primary labeled glutamate was identified as E681 in TM8 (252). Alper and coworkers showed that mutagenesis of this residue (E699) in mouse band 3 to glutamine has very similar functional effects (253, 254), providing further evidence that E681, when protonated, converts band 3 from a monovalent to a divalent anion transporter, and the H^+^ bound to E681 is cotransported with $SO_{4}^{2-}$. Cysteine scanning mutagenesis of TM8 showed that the E681C mutant has no transport activity (255); the E681Q mutant has very low transport activity (165).

In the crystal structure (18), the E681 side chain is adjacent to the substrate binding pocket (Fig. 6). On the other side of the substrate binding pocket is the side chain of R730, a probable target of arginine-selective reagents that inhibit red cell anion transport (256–260). The charge configuration at the binding pocket therefore consists of the positive charge on R730 and the NH_2_-terminal ends of TM3 and TM10 helical dipoles and the negative charge on E681 and substrate anion. Uncharged but polar side chains near the substrate site are S465 in TM3 and S725, T727, and T728 in TM10 (Fig. 6).

The normal anion translocation event is nearly electroneutral (118, 239, 261), but if the charge on E681 is removed by either WRK/$BH_{4}^{-}$ (251) or mutagenesis (253, 254), Cl^−^/$SO_{4}^{2-}$ exchange becomes electrogenic and has altered pH dependence. This is consistent with the idea that, in the normal translocation event, the negative charge on E681 moves with the substrate ion relative to the transmembrane electric field, balanced by the positive charge on R730 and the positive ends of the TM3 and TM10 helix dipoles.

## ASSOCIATIONS OF BAND 3 DIMERS

### Band 3 Tetramer and Higher Oligomer

A significant fraction of purified band 3 in detergent is tetrameric (171, 262, 263), and the ankyrin-bound form of band 3 is tetrameric (264). Ankyrin contains two folds that could each bind one band 3 dimer (265). Other evidence for tetrameric band 3 is that CuP forms some covalent tetramers in addition to dimers (177, 266). Dimers cross-linked by BS^3^ can form noncovalent tetramers that are stable in SDS at moderate temperatures, indicating that there are interactions between adjacent dimers (267). Clusters of band 3 larger than tetramer are associated with a variety of pathophysiological conditions, including oxidative stress, hemoglobin denaturation, and cell senescence (268–273).

### Glycophorin A

There are many indications that band 3 and glycophorin A (GPA) interact closely (274). Antibodies against GPA reduce band 3 rotational mobility (275). Coexpression with GPA enhances band 3 expression in *Xenopus* oocytes (276, 277). GPA forms a complex with band 3 in the ER of K562 cells, but knockdown of GPA expression does not decrease surface expression of band 3 in these cells (278). A Wright blood group antigen is formed by GPA and E658 of band 3 (279). Red cells that lack GPA, but not glycophorin B (GPB), have ∼50% lower anion transport rates (228, 229). Different regions of GPA are associated with increased band 3 trafficking and increased anion transport (280, 281). Glycophorin Mur, the product of homologous recombination of GPA and GPB, enhances band 3 expression (282, 283).

### Glycolytic Enzymes and Deoxyhemoglobin

Glyceraldehyde-3-phosphate dehydrogenase (GAPDH) binds to the band 3 cytoplasmic domain and is inactive when bound (284, 285). Aldolase (286), phosphofructokinase (287), and deoxyhemoglobin (288–290) also bind to the cytoplasmic domain. Deoxyhemoglobin binding interferes with glycolytic enzyme binding (10, 11). The binding of these proteins is reversed by phosphorylation of two tyrosine residues (Y8 and Y21) near the NH_2_ terminus (291).

The significance of glycolytic enzyme binding to band 3 has been questioned (292) because binding to membranes is not observed at physiological ionic strength. Later work, however, demonstrated GAPDH binding to band 3 in intact cells (293, 294). More recent confocal images show that GAPDH, aldolase, phosphofructokinase, pyruvate kinase, and lactate dehydrogenase are mainly membrane associated in a glycolytic enzyme complex anchored to band 3 in intact cells (295, 296).

### Peroxiredoxin

Peroxiredoxin-2 (Prx2), is a very abundant cytosolic protein that scavenges peroxides in red cells (297) and binds to the band 3 cytoplasmic domain (298). It is not known whether band 3 binding is involved in the role of Prx2 (also known as calpromotin) in Ca^2+^-sensitive K^+^ transport in red cells (299).

### Carbonic Anhydrase II

Vince and Reithmeier (300, 301) used immunofluorescence, immunoprecipitation, and solid-phase binding assays to demonstrate carbonic anhydrase II (CAII) binding to an acidic sequence (D887ADD) near the band 3 COOH terminus. This work led to the idea that bound CAII acts in concert with band 3 to form a transport metabolon that facilitates capillary CO_2_ exchange (302–307). Transport metabolons have been proposed for other acid-base transporters (308–312) and CO_2_ channels (283). The band 3-CAII metabolon is discussed further below in connection with ongoing questions.

## BAND 3 MOBILITY

### Lateral Mobility

Electron microscopy showed that erythrocyte intramembranous particles, including band 3, have lateral mobility (313). In red cells fused with Sendai virus or polyethylene glycol (PEG), surface proteins (mainly band 3) prelabeled with fluorescein can diffuse laterally into the membranes of cells that had not been labeled (314). Fluorescence photobleaching recovery (FPR) experiments demonstrated subpopulations of band 3 with different lateral mobilities (315, 316).

The lateral mobility of band 3 is largely constrained by the spectrin meshwork (7), but recent single-particle experiments have shown that, in relatively rare events (∼3/s), band 3 can “hop” to the adjacent element of the meshwork (317). Very mild trypsin digestion of the band 3 cytoplasmic domain without major disruption of spectrin increases the “hop” rate, consistent with the idea that the cytoplasmic domain, in copies of band 3 not directly bound to the membrane skeleton, hinders movement of band 3 across the lateral cytoskeletal barrier.

The trajectories of single band 3 molecules labeled by quantum dots indicate that in normal red cells there are immobile, constrained, and freely diffusing populations (318). The same methodology applied to mouse erythrocytes showed that ∼40% of band 3 is attached to ankyrin, 33% attached to adducin, and ∼27% not attached, with heterogeneity within each population (319). Surprisingly, the lateral diffusion of GPA measured with quantum dots is distinct from band 3 (320) and indicates that the association between GPA and band 3 in mature red cells may be relatively weak.

### Rotational Mobility

Transient dichroism of band 3 labeled with eosin isothiocyanate or iodoacetamido-eosin showed that ∼40% of band 3 has restricted rotational mobility; when either the cytoplasmic domain is cleaved or cytoskeletal proteins are extracted, the mobility of this population increases (321, 322). The rotational mobility of E5M-labeled band 3 reveals heterogeneity, with ∼20% of the population rotating rapidly (correlation time 50 ± 30 µs) and a larger population (variable estimates) with ∼1-ms correlation times (323–327). As is true of lateral diffusion, rotational diffusion is temperature dependent (323) and restricted by binding of ankyrin (326, 327) or hemichromes (324). Band 3 rotation measured with an electron paramagnetic label is consistent with a homogeneous population of band 3 dimers (328, 329).

### Do Mobile and Immobile Subpopulations of Band 3 Have Different Transport Properties?

Evidence that mobile and immobile populations of band 3 have the same transport properties comes from work with band 3 Prague, which is misfolded and not present in mature red cells (330). In red cells of heterozygotes for band 3 Prague, the amount of band 3 and the $SO_{4}^{2-}$ flux are both decreased by 40%. Therefore, each copy of normal band 3 has the same transport activity whether it is in normal red cells or the cells of band 3 Prague heterozygotes. The fraction of laterally mobile band 3 is much lower in the heterozygotes than in normal red cells (330). This suggests that mobile and immobile band 3 have indistinguishable transport properties in normal red cells and that whatever internal molecular motions are associated with transport are independent of lateral diffusion of the whole protein.

## SUMMARY OF BAND 3 COMPLEXES

Starting with the classic work of Steck (284), there have been numerous models of the arrangement of red cell membrane proteins (7, 8, 331–334). In mature normal human red cells, band 3 can exist in the following states:

1. Untethered dimer. Based on the population with the fastest rotational diffusion and lateral diffusion within a cytoskeletal corral, ∼20% of band 3 is in the form of untethered dimer. GPA and Prx2 are sufficiently abundant to be associated with all forms of band 3, including the untethered dimer (7), but GPA does not appear to be associated with the untethered fraction of band 3 (320).
2. Ankyrin complex, consisting of one band 3 tetramer, one ankyrin molecule, and several other proteins (7). About 40% of the band 3 polypeptides are in this complex. Other integral proteins include GPA, CD47, Rh proteins, GPB, and Landsteiner–Wiener antigens (7, 335, 336). Peripheral proteins include band 4.2 (331, 337) and the glycolytic enzyme complex (295).
3. Adducin complex (338, 339), at the spectrin-actin junction. The adducin complex is larger than the ankyrin complex and includes band 4.1, dematin, stomatin, and many other integral and peripheral proteins (7, 333, 340), including the glucose transporter GLUT1 (341). Some of the components of the complex are far less abundant than band 3; therefore, the composition of the junctional complex is heterogeneous (see Ref. 7). Red cells that are deficient in stomatin have about half the Cl^−^/$\mathrm{HC}O_{3}^{-}$ exchange activity of normal red cells (342); not enough is currently known about the interaction between band 3 and stomatin for a detailed interpretation of this result.

The linear decrease in transport with stilbenedisulfonate binding (70, 76, 225–230) is consistent with the idea that each copy of band 3 has the same transport properties, irrespective of the complex it is in. However, it is still possible that band 3 in, say, the adducin complex has a different transport rate than other copies of band 3. If DIDS binding affinity is the same in all copies of the protein, then there will be a linear decrease in transport with increasing DIDS binding, even if subpopulations have different transport properties. There is no evidence for different transport properties of band 3 in the above states of association, but the possibility cannot be ruled out.

## REGULATION OF BAND 3-MEDIATED ANION TRANSPORT

### Lack of Physiological Regulation of Red Cell Band 3

The physiological function of $\mathrm{HC}O_{3}^{-}$ transporters is pH homeostasis. Some $\mathrm{HC}O_{3}^{-}$ transporters, including AE2 and AE3, are acid loaders (base extruders), because the inward Cl^−^ gradient exceeds the inward $\mathrm{HC}O_{3}^{-}$ gradient in most cells (1, 2, 4). The regulation of AE2 has been studied extensively by Alper and coworkers. In the physiological pH range, a higher pH activates AE2, resulting in a negative feedback loop that limits cytoplasmic alkalinization (343). Both intracellular and extracellular pH affect transport in the physiological range (344), and both the cytoplasmic and membrane domains are involved in the regulation (345). AE2 is also activated by NH_3_/$NH_{4}^{+}$ (346) and by hypertonic conditions (347). Although regulatory regions have been identified in AE2 (344, 348–351), the detailed regulatory mechanisms are not yet fully understood.

In contrast to AE2, anion exchange by red cell band 3 is not strongly affected by pH in the physiological range (224, 352, 353). The only physiological regulation of AE1 by pH is a moderate upregulation of kAE1 expression in response to an acid load (354–356). Red cell Cl^−^ transport is very similar in intact red cells and resealed ghosts if Donnan effects on ion distribution are taken into account (93), consistent with the idea that band 3 transport does not require continuous maintenance by cytoplasmic constituents. There is evidence that red cell band 3-mediated oxalate transport is stimulated by increased Ser/Thr phosphorylation following okadaic acid treatment (357, 358), but our laboratory found no effect of okadaic acid on oxalate transport (227). ATP depletion of red cells causes a 30–50% reduction in anion transport (227, 359, 360); the mechanism of this effect is not known, but in any case, the effect is moderate.

The lack of major regulation of red cell band 3 transport during the normal life of the red cell is consistent with its role in capillary CO_2_ exchange (Fig. 2). When the cell arrives in a pulmonary or systemic capillary, band 3 must exchange Cl^−^ for $\mathrm{HC}O_{3}^{-}$ very rapidly in response to the sudden change in $\mathrm{HC}O_{3}^{-}$ gradient. After the cell leaves the capillary, there is no reason to downregulate transport, because there is very little postcapillary driving force for Cl^−^/$\mathrm{HC}O_{3}^{-}$ exchange (361). If transport were downregulated after leaving either a pulmonary or systemic capillary, there would be very little time (<<1 s) to reactivate it when the cell next arrives in a capillary. It is more likely that, for most of the lifetime of the normal red cell, band 3 transport is constitutively active.

### Effects of Oxidative Stress, Thiol Status, and Tyrosine Phosphorylation

Although the main transport function of band 3 is not up- and downregulated under physiological conditions, transport activity can be reduced by oxidative stress and other pathophysiological conditions (12, 13, 17, 268, 269, 362–364). The literature on band 3 and red cell oxygen status is too extensive to cover in detail here; the discussion below is focused on effects of oxidative stress on band 3-mediated anion transport.

All 5 cysteine residues in human band 3 can be replaced with serine without major loss of transport function (365). Casey and coworkers (255, 366–368) have used cysteineless band 3 for informative studies of topology and function by cysteine scanning mutagenesis, as reviewed by Reithmeier et al. (20). Despite the fact that band 3 has no cysteine residues that are necessary for transport, treatment with *N*-ethylmaleimide (NEM) or oxidative stress inhibits red cell anion transport to varying degrees (369–375).

A probable connection between thiol status and transport is tyrosine phosphorylation (363, 376). Sites of tyrosine phosphorylation on band 3 include Y8 and Y21 near the NH_2_ terminus; Y359 in the linker between membrane and cytoplasmic domains; and Y904 near the COOH terminus (377–380). There is evidence for a role of Y359 and Y904 phosphorylation in kAE1 trafficking (381, 382), but neither is necessary for anion transport; band 3 can be cleaved by trypsin adjacent to Y359 without major effect on transport (184, 185), and transport is normal in band 3 Walton, which is missing residues 901–911 (381).

**Figure 7.**
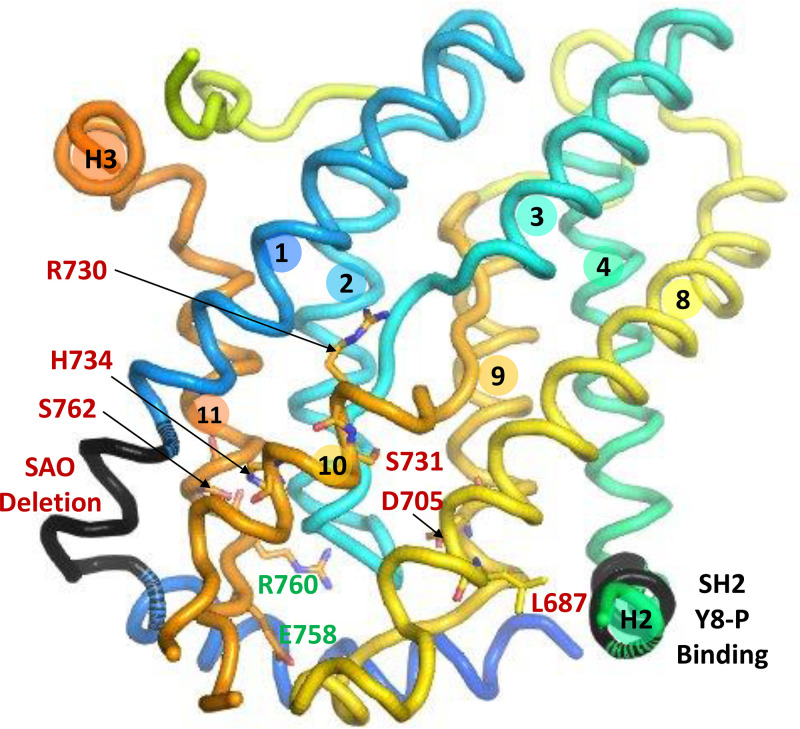
Band 3 core domain, viewed from the gate/dimerization domain, showing the location of the Southeast Asian ovalocytosis (SAO) deletion (411) and the SH2 sequence that binds cytoplasmic domain phosphorylated at Y8 with transport inhibition (386). Sites of human point mutations that cause increased cation leak and major inhibition of anion transport are labeled in red: L687P, D705Y, S731P, H734R (428), R730C (429, 430), and S762R (431). Mutation G796R (not shown), which also causes cation leak and anion transport inhibition (430, 432), is in the TM12 of the gate domain, facing R730. Two sites (E758K and R760Q) of mutations that cause increased cation leak but do not strongly affect anion exchange are labeled in green.

Phosphorylation of Y8 and Y21 in red cells has been characterized in connection with regulation of metabolism and oxidative damage (291, 380, 383–385). Y8 phosphorylation, most likely by Syk, results in the binding of the phosphorylated cytoplasmic domain to an SH2 sequence (G509–R514) in cytoplasmic helix H2 linking core and gate of the membrane domain (Fig. 7); the binding strongly inhibits anion transport (386). This is the most direct connection to date between the cytoplasmic domain and anion transport and challenges the long-held view that anion transport is independent of the cytoplasmic domain. In addition to inhibiting transport, phosphorylation of Y8 causes displacement of glycolytic enzymes and release of ankyrin (386).

It is not yet clear what physiological or pathophysiological conditions result in Y8 phosphorylation and transport inhibition. Red cell protein tyrosine phosphatase (PTP) activity is normally high (363, 371, 376, 387–389), and it is possible that Y8 is mainly unphosphorylated during most of the normal red cell lifetime (see further discussion below).

## ANION TRANSPORT AND NATURALLY OCCURRING HUMAN *SLC4A1* MUTATIONS

*SLC4A1* genetic variants are associated with hereditary spherocytosis (HS), stomatocytosis (HSt), or distal renal tubular acidosis (dRTA) (16, 20, 390–394). Many dominant *SLC4A1* mutations associated with HS cause band 3 deficiency resulting from misfolding and protein instability; band 3 is such a major part of red cell architecture that even a moderate deficiency causes shape changes and/or fragility (16, 390, 395, 396). There is usually no renal phenotype because heterozygotes produce and target sufficient amounts of normal kAE1.

*SLC4A1* dRTA variants usually have trafficking defects that result in kAE1 mistargeting (230, 393, 397, 398). In dominant dRTA, the protein is folded and inserted sufficiently to form heterodimers with normal band 3, but the heterodimer has impaired trafficking that reduces the amount of normal functional kAE1 (398, 399). For most dRTA variants, the trafficking defect is rescued in red cells by coexpression of GPA (274, 400), although there are variants that cause both dRTA and HS (230).

It is beyond the scope of this review to discuss the pathophysiology of HS, HSt (401–403), or dRTA (404, 405), all of which can have causes other than *SLC4A1* mutations. The focus here is on transport by band 3 variants in red cells or heterologously expressed.

### Memphis: K56E

Mueller et al. (406) first described a human band 3 variant with reduced electrophoretic mobility of the 60-kDa chymotryptic fragment as a result of a K56E substitution (407, 408). The mutation has at most minor functional effects; band 3-mediated phosphoenolpyruvate transport in red cells from homozygotes is ∼20% lower than normal (409).

### S725R Variant

Yang et al. (410) recently described a band 3 variant, S725R, in which homozygotes have band 3 deficiency, anemia, and dRTA. The S725R protein trafficks to the cell surface when expressed in HEK cells, but it has no transport activity, whereas the S725A protein has normal transport. S725 is close to E681 in the crystal structure (Fig. 6), and the disruption caused by arginine substitution likely results from the arginine side chain interacting with the E681 side chain (410).

### Southeast Asian Ovalocytosis

The band 3 Southeast Asian ovalocytosis (SAO) variant has the Memphis mutation and a deletion of residues 400–408 covering the COOH-terminal end of cytoplasmic helix H1 and the NH_2_-terminal 4 residues of TM1 (411) (Fig. 7). Red cells of SAO heterozygotes are abnormally rigid and resistant to invasion by *Plasmodium falciparum* (412). The SAO mutation does not cause dRTA, but additional mutations causing dRTA are sometimes found in SAO heterozygotes (394). It was previously believed that homozygosity for the SAO variant is lethal, but SAO homozygotes have survived with careful management (413–416).

The SAO deletion is expected to have a major conformational effect on TM1 (417–419), but the protein is nonetheless stably associated with the membrane. Nearly half the band 3 in heterozygotes is SAO band 3, but SAO band 3 does not bind stilbenedisulfonates and has abnormal mobility (412, 420–422). Normal and SAO band 3 can form heterodimers (423) and higher heterooligomers (419). Heterodimers also form between normal and SAO kAE1 expressed in HEK293 cells (424). Anion transport in red cells of SAO heterozygotes, including those with dRTA mutations, is close to or slightly less than half of normal (226, 423, 425, 426). This is consistent with the idea that the normal copy of band 3 in a SAO heterodimer can transport anions, but possibly more slowly than normal.

### HSt Mutations with Very Low Anion Transport and Elevated Cation Transport

Red cells of SAO heterozygotes have elevated Na^+^ and K^+^ permeability, especially at low temperatures (427). Several band 3 point mutations associated with HSt also have strongly reduced anion transport and elevated cation permeability: L687P, D705Y, S731P, H734R (428), R730C (429, 430), S762R (431), and G796R (430, 432). Severe anion transport disruption by these mutations is consistent with the crystal structure (Fig. 7). R730 and S731 are very close to the likely substrate binding site. L687 is near the H2 helix connecting core and gate. The S762 and H734 side chains are close to each other in the crystal structure; an arginine substitution for either would disrupt the TM10/TM11 packing near the substrate site. D705 is near the cytoplasmic end of TM9 and is not as close to the substrate site. G796 is in TM12 of the gate (not shown in Fig. 7), facing the NH_2_-terminal end of the TM10 helix near the substrate binding pocket in the core.

### Mutations with Nearly Normal Anion Transport and Elevated Cation Transport

Band 3 with E758K substitution is associated with spherostomatocytosis and somewhat increased cation permeability but has near-normal anion transport when coexpressed with GPA in oocytes (433). The increased cation flux induced by expression of E758K band 3 is not dependent on GPA, raising the possibility that the ^86^Rb^+^ flux is by way of a separate pathway. Red cells from a heterozygote for H734R band 3 also have elevated cation transport by pathways other than band 3 (434).

Red cells from heterozygotes for the dominant HS mutation R760Q have moderately increased cation permeability and ∼75% of normal $SO_{4}^{2-}$ transport activity (428), indicating that the variant protein carries out substantial anion transport. R760Q AE1 expressed in *Xenopus* oocytes has normal Cl^−^/$\mathrm{HC}O_{3}^{-}$ exchange activity (435). The variant does, however, have a trafficking defect (436) and is difficult to detect in red cells of R760Q heterozygotes (437). E758 and R760 are on the cytoplasmic end of TM11 in the core (Fig. 7).

Several kAE1 variants associated with dRTA (R589H/C, G609R, S613F, G701D, D905dup, E906X/K, G609R) have normal or near-normal anion transport activity (425, 438), and some (R589H, G609R, S613F, G701D) have elevated stilbenedisulfonate-sensitive ^86^Rb permeability at 0°C in *Xenopus* oocytes (438). The lack of effect of these mutations on anion transport activity is consistent with the crystal structure; the substitutions are not close to the anion binding site. There is no clear pattern to the substitutions that cause increased cation permeability.

It is important to point out that cation transport in band 3 variants is extremely slow compared with normal anion exchange. Ellory et al. (435) calculated that in the cation-transporting band 3 mutants the number of cations transported per copy of band 3 is only ∼1 ion/s. There is no known connection between cation transport in these variants and the K^+^ transport through red cell band 3 in media of low ionic strength (121).

### HS Mutation H834P

The variant H834P results in HS (395) with impaired folding and trafficking to the plasma membrane (436). Chemical modification with diethylpyrocarbonate (439) or site-directed mutagenesis (368, 440, 441) indicates that H834 is important for transport. In the crystal structure it is located near the cytoplasmic end of TM13.

### Band 3 HT: P868L

The band 3 variant P868L is associated with acanthocytosis and is known as band 3 HT, because it has a higher maximal anion transport rate than normal (442, 443). The variant is also labeled with H_2_DIDS less readily. The reason transport is higher in P868L band 3 is not known but it may result from a change in the core-gate connection; P868 is in TM13 of the gate, close to where cytoplasmic helix H3 connects core TM4 to gate TM5.

### Mutations Causing Band 3 Null Phenotype: Coimbra, Vienna

The mouse band 3 null phenotype, with absence of band 3 in both red cells and kidney, produces numerous abnormalities, including spherocytosis and severe anemia, GPA deficiency (444), dyserythropoiesis (445), increased Ca^2+^ leak and cell death (446), but relatively normal membrane skeleton architecture (447). The null phenotype is similar in cattle (448).

There have been a few examples of humans with the band 3 null phenotype, resulting from homozygous band 3 Coimbra (V488M) (449) or Vienna (S477X) (450). The human band 3 null phenotype includes spherocytic anemia, dRTA at 3 mo, and dyserythropoiesis; regular transfusions and oral bicarbonate therapy for dRTA have made it possible for band 3-null patients to survive into childhood (450).

## ONGOING QUESTIONS ABOUT BAND 3

### Structure and Interaction with Other Proteins

In his excellent review of the red cell membrane skeleton, Lux (7) identified several unanswered questions about band 3, the first of which is “What is the overall structure of band 3?” Although crystal structures of both the membrane and cytoplasmic domains of band 3 are known, there is still uncertainty about the structure of the intact protein. The cross-linking experiments of Rivera-Santiago et al. (187) resulted in a model with extensive contacts between the two domains. However, several residues can be cross-linked to multiple partners, indicating that the association between membrane and cytoplasmic domains has some flexibility. Molecular dynamics simulations by De Vecchis et al. (451) generated alternative models, the most compact of which differs from that of Rivera-Santiago et al. (187). Further modeling as well as direct measurement of binding interactions between the two domains can potentially improve our understanding of the structure of intact band 3.

Another unanswered structural question is the extent of conformational lability of amino acids 812–830, which in the crystal structure (18) include parts of cytoplasmic helices H4 and H5 between TM12 and TM13 (see Fig. 3 and Fig. 4). A peptide consisting of residues 812–827 was originally shown by Kay et al. (452) to inhibit binding of senescent cell IgG to senescent red cells, indicating that the sequence is extracellular. Cysteine scanning mutagenesis and labeling of AE1 expressed in HEK 293 cells also indicated that these residues are extracellular (366, 368). Badior and Casey (453) recently reexamined the topology of this sequence in AE1 expressed in HEK cells and found that this sequence is mainly cytoplasmic, consistent with the crystal structure, but it can reorient transiently to a conformation in which it is exposed to the extracellular medium. Binding of extracellular IgG can potentially trap this rare conformation. Over time, enough band 3 could become bound with IgG to mark the cell as senescent. The reorientation of residues 812–830 could therefore serve as the molecular clock for erythrocyte senescence (453). This new idea will likely be tested in future experiments and simulations.

### Transport Mechanism: Rocking versus Elevator

**Figure 8.**
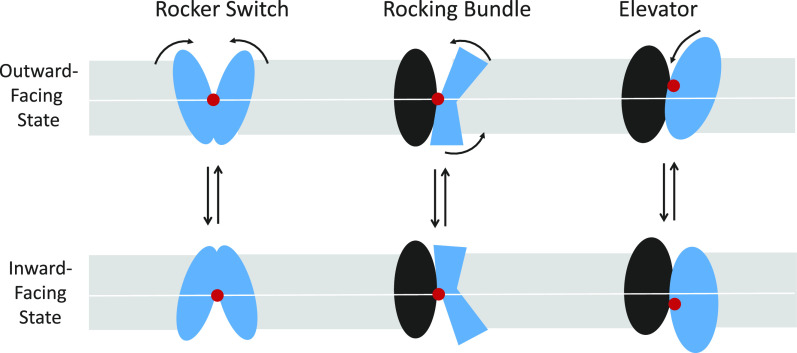
Schematic representation of possible mechanisms (rocker switch, rocking bundle, and elevator) for alternating access transport (454, 510, 534). For band 3 rocking bundle and elevator mechanisms, the core domain is blue and the gate domain is black.

There are many unanswered questions about the molecular mechanism and the integrative physiology of band 3-mediated anion transport. Possible mechanisms for alternate access transporters include rocker switch, rocking bundle, and elevator (454, 455) (Fig. 8). The rocking-type mechanisms have a nearly stationary substrate binding site (red circle in Fig. 8). In elevator-type mechanisms, a domain of the protein, with bound substrate, moves relative to a fixed scaffolding domain (Fig. 8, *right*). Yeast SLC4 transporter Bor1 appears to have a rocking bundle mechanism (456), but other related transporters, including bacterial UraA (457) and *Arabidopsis* Bor1 (458), are believed to use an elevator-like mechanism (459).

For red cell band 3, repeat swap homology modeling (460) predicts an elevator-type mechanism that is consistent with many known experimental properties of red cell anion exchange (461). The elevator mechanism is also consistent with the inhibitory effect of phosphorylated Y8 binding to the SH2 domain in the H2 helix connecting core and gate (386) (Fig. 7); this helix participates in the translocation event in the elevator model.

### Transition State

A genuine understanding of the transport mechanism will require knowledge not only of the structure of the inward-facing state but also of the transition state between inward- and outward-facing structures. Insights into the nature of the transition state could help explain some poorly understood properties of band 3-mediated transport:

- Variations in transport rates among different substrates. Little is currently known about substrate anion binding to the transition state and whether band 3 forms an occluded state, as is known to exist for UraA (462). Information about anion binding to the transition state (107, 110, 111) could help explain the huge variations in transport rates (Table 1) among anions that appear to have similar affinities for the transport site.
- High rate of Cl^−^ and $\mathrm{HC}O_{3}^{-}$ transport. The band 3-mediated Cl^−^/Cl^−^ exchange flux at body temperature is ∼50,000 ions/s per band 3 polypeptide (224). With symmetric translocation, the forward and reverse unimolecular rate constants would both be 100,000/s (Fig. 2). With asymmetric translocation (94), one of the rate constants is even higher. There is currently no explanation for such high translocation rate constants.
- High activation enthalpy and nonlinear Arrhenius plots. The high Cl^−^/$\mathrm{HC}O_{3}^{-}$ exchange rate is especially remarkable considering that the activation enthalpy is 20 kcal/mol at 25–38°C and even higher at low temperatures (51, 224). The high rate constant with high activation enthalpy indicates a large positive entropy of activation (463, 464). It is not known how the transition state could have a high entropy and why nonlinear Arrhenius plots are observed for exchange of halides, $\mathrm{HC}O_{3}^{-}$, and other oxyanions (39, 224, 464, 465).
- High activation volume. Band 3-mediated anion exchange has a large dependence on hydrostatic pressure and therefore a high activation volume, indicating that the rate-limiting event results in expansion of the protein-anion complex by 0.15 L/mol (466). There is currently no explanation for the high activation volume.
- Low “slippage” rate. The rate constant for translocation without substrate (“slippage”) is extremely low (115, 116). In contrast, for SLC4 Na^+^-$\mathrm{HC}O_{3}^{-}$ cotransporters (1–3), the catalytic cycle presumably includes reorientation of the protein without substrates. The factors governing the transition state of the empty transporter (forbidden for exchangers; required for cotransporters) is a major unanswered question for SLC4 transporters.
- Self-inhibition. It is well established experimentally that high concentrations of halides inhibit halide exchange (83, 89, 90). The molecular basis for self-inhibition is not known and may or may not involve the transition state.
- Lipid effects. Molecular dynamics simulations predict an annular lipid layer and cholesterol enrichment at the band 3 dimer interface (178), but there is currently no explanation for how cholesterol enrichment lowers the rate of band 3-mediated anion transport (467–469).
- Roles of K539 and K851, if any, in transport. Mutagenesis of the H_2_DIDS-cross-linked residues K539 and K851 lowers the affinity for reversible binding of stilbenedisulfonates but does not prevent Cl^−^ transport (220). BS^3^, in addition to the intermolecular cross-link of the TM5-TM6 loop, forms an intramolecular cross-link, probably between K539 and K851 (176, 177). The intramolecular cross-link results in partial transport inhibition with altered pH dependence and apparently lower affinity for the self-inhibitory site (233), but these effects are not understood.

### Role of Cytoplasmic Domain and Oxidative Stress in Transport

Oxidative stress produces many changes in subpopulations of band 3, including clustering, release of ankyrin, and diminished anion transport (12, 363, 371, 384, 388, 389, 470–472). The mechanism for these effects is likely to include PTP inhibition, phosphorylation of Y8, and binding of the cytoplasmic domain to an SH2 domain on the membrane domain (386). Further work is needed to understand the many different physiological, pathophysiological, and pharmacological conditions affecting the tyrosine phosphorylation status of band 3 (362, 473–479).

### Transport Metabolon

As described above, there are several lines of evidence that CAII binds to an acidic sequence near the band 3 COOH terminus (300, 301). There is also evidence to the contrary; solid-phase binding assays and surface plasmon resonance do not detect CAII binding to band 3 peptides containing the expected CA binding sequence (480, 481), and the two proteins do not detectably associate as measured by FRET or immunoprecipitation (482). It is not clear why binding studies in different laboratories disagree; part of the reason could be the relatively low binding affinity (311).

In addition to binding studies, there is a large body of evidence that CAII association with band 3 has functional consequences in mammalian cells or *Xenopus* oocytes expressing normal and mutated AE1 and CAII (302–307). These studies showed in several different ways that association of band 3 with catalytically active CAII is required for full transport activity, measured as the rate of intracellular pH change after step increases or decreases in extracellular [Cl^−^].

Although there is extensive evidence for a functional band 3-CAII metabolon in expression systems, it is also well established that, in red cell band 3, CAII is not necessary for full $\mathrm{HC}O_{3}^{-}$ transport activity, defined as a complete cycle of binding, translocation, and release of $\mathrm{HC}O_{3}^{-}$ in both directions. To measure [^14^C]$\mathrm{HC}O_{3}^{-}$/$\mathrm{HC}O_{3}^{-}$ exchange, the activity of CA must be inhibited to minimize [^14^C]CO_2_ flux. Even in the presence of strong CA inhibition, band 3-mediated $\mathrm{HC}O_{3}^{-}$ self-exchange is very rapid, comparable to the Cl^−^ self-exchange (76, 352). Another method for measuring $\mathrm{HC}O_{3}^{-}$ transport in red cells is ^18^O exchange (482–484). Al-Samir et al. (482) showed that red cells from donors lacking CAII have normal $\mathrm{HC}O_{3}^{-}$ permeability determined by this method, further indication that CAII does not accelerate band 3-mediated $\mathrm{HC}O_{3}^{-}$ transport in red cells. Al-Samir et al. (482) also presented mathematical modeling showing that pulmonary capillary CO_2_ exchange is more efficient with uniform CA in the cytosol rather than concentrated at the membrane.

In summary, it remains possible that there is a functionally significant red cell band 3-CAII metabolon, but a viable metabolon model needs to demonstrate why concentrating CA at the inner membrane surface is more efficient than distributing CA in close proximity to hemoglobin, the main H^+^ buffer (482). Future metabolon models also need to explain how bound CAII can preferentially channel $\mathrm{HC}O_{3}^{-}$ to and from band 3 but not interfere with the exchange of Cl^−^ between cytosol and the same inward-facing transport site. The same consideration applies to any proposed metabolon involving a transporter that has more than one substrate, i.e., how to channel one substrate and not get in the way of the other.

### Other Unanswered Questions about Capillary Gas Exchange

**Figure 9.**
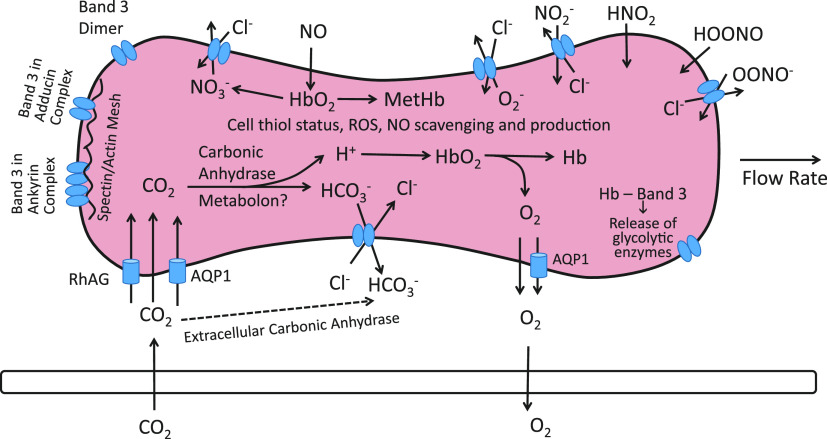
Events taking place during exchange of CO_2_ and O_2_ in a systemic capillary. CO_2_ enters the cell by a combination of solubility diffusion and transport through aquaporin 1 (AQP1) and Rh associated glycoprotein (RhAG) (535–537). Cytoplasmic CO_2_ is hydrated by carbonic anhydrase, either bound to band 3 as a metabolon (303) or in the cytosol (482). The $\mathrm{HC}O_{3}^{-}$ formed is transported outward in exchange for Cl^−^ on subunits of the band 3 dimer or tetramer, either as an untethered dimer or in complexes with ankyrin or adducin. The other protein components of these complexes are not shown. The acid formed from CO_2_ hydration is buffered by hemoglobin, which is simultaneously releasing O_2_ in response to O_2_ efflux from the cells by a combination of solubility diffusion and transport through AQP1 (538). In parallel with CO_2_ uptake and O_2_ release, there is scavenging and production of nitric oxide (NO) and transport of NO metabolites either inward or outward on band 3 or by diffusion of undissociated acids (see text). Other processes (not shown) are carbamate formation and Cl^−^ binding to hemoglobin. There is also some hydration of CO_2_ in the plasma.

Figure 9 is an updated version of Fig. 1 showing the events that take place during capillary CO_2_ exchange. Much more is now known about anion transport through band 3, but there is more to be learned about how band 3-mediated transport contributes to capillary CO_2_ and O_2_ exchange, including:

- Timing of the effects of deoxygenation on hemoglobin binding to band 3. It is clear that deoxygenation causes increased binding of hemoglobin to band 3 and changes in metabolism (11). It is not known, however, how these changes in metabolism affect the cell during the normal repeated cycling of oxygenation and deoxyhemoglobin. How much deoxygenation, and over what time period, is needed to produce a significant effect on red cell metabolism? This question does not relate directly to anion transport, but it is part of the overall dynamics of the status of band 3 during capillary gas exchange.
- Extracellular carbonic anhydrase. Intracellular carbonic anhydrase activity is definitely much higher than that on the surface of endothelial cells in pulmonary and systemic capillaries (485, 486), CAIX bound to the extracellular surface of band 3 (487), or plasma CA resulting from red cell lysis. One role of extracellular CA is to minimize postcapillary pH transients (361). The quantitative contribution of extracellular CA to capillary CO_2_ exchange may be low but is not known.
- Effect of reduced red cell Cl^−^/$\mathrm{HC}O_{3}^{-}$ exchange. All vertebrates except lamprey (488) have high rates of red cell Cl^−^/$\mathrm{HC}O_{3}^{-}$ exchange, and modeling predicts that inhibition of anion exchange should lower the CO_2_-carrying capacity of blood (29, 36). In agreement with this prediction, inhibition of band 3 by right atrial infusion of 4,4′-dinitrostilbene-2,2′-disulfonate (DNDS) lowers pulmonary CO_2_ excretion by ∼15% in dogs (489). Moderate (50%) inhibition of band 3-mediated transport does not appear to have a measurable effect on blood CO_2_ transport and pulmonary gas exchange, as indicated by SAO heterozygotes (without dRTA) who have normal arterial blood gas values and ∼50% of normal band 3 transport (490). In SAO homozygotes and band 3-null humans, mice, and cattle, the effects of lack of red cell anion exchange are complicated by the coexisting anemia and dRTA. The quantitative effects of graded decreases in red cell anion exchange, without other effects, are not known, either experimentally or theoretically. It is also not known whether a moderate reduction in red cell anion exchange would affect CO_2_ excretion during exercise.
- Possible physiological role of band 3 transport of nitric oxide (NO) metabolites. In the past 20 years there has been an explosion of information on the role of the red cell in NO metabolism. The red blood cell has a role in both the production and scavenging of NO (491, 492), and the NO metabolites nitrate ($NO_{3}^{-}$), nitrite ($NO_{2}^{-}$), and peroxynitrite (OONO^−^) are all transported by band 3 (Table 1). NO reacts with oxyhemoglobin to produce methemoglobin and $NO_{3}^{-}$, which is transported by band 3 nearly as rapidly as $\mathrm{HC}O_{3}^{-}$ (493); it is not known whether rapid $NO_{3}^{-}$ transport is of importance for NO scavenging. Both $NO_{2}^{-}$ and OONO^−^ can be transported by diffusion of the cognate weak acids as well as by band 3 (494–497). It is possible that band 3-mediated transport of these solutes is of secondary importance, but an integrative understanding of red cell NO metabolites and their relationship with blood flow regulation (491, 492, 498–500) should include consideration of the possible role of band 3-mediated transport of these metabolites and of $O_{2}^{-}$.
- Intracellular diffusion in red cells during capillary O_2_ and CO_2_ exchange. Imaging and mathematical modeling have produced new insights into the processes of intracellular diffusion of CO_2_, H^+^, $\mathrm{HC}O_{3}^{-}$, and non-$\mathrm{HC}O_{3}^{-}$ buffers in red cells (353, 484, 501, 502). Intracellular diffusion of O_2_ has long been known to be facilitated by hemoglobin (Hb) (503), but recent single-cell imaging studies indicate that intracellular O_2_ diffusion is slower than previously believed (504). The next level of understanding the intracellular diffusion in red cells during capillary exchange should include the relationships among the intracellular gradients of CO_2_, H^+^, buffers, Cl^−^, dissolved O_2_, hemoglobin, and deoxyhemoglobin, and how these gradients are affected by HbO_2_ dissociation rates (505), carbonic anhydrases I and II (506), Cl^−^ binding to hemoglobin (507), and carbamate formation (508). A better understanding of all the diffusion processes would also help resolve the metabolon controversy.

## SUMMARY

Perhaps the most important lesson that has emerged from decades of research on red cell band 3 is that even relatively simple systems are still complex, and not all the answers are in, despite the tremendous progress that has been made in the study of band 3. It is fair to ask whether it is still useful to continue to work on a system that is has been as thoroughly studied as band 3. I think the answer is that band 3 is still a great experimental system for applying new methods, as exemplified by the cross-linking study of Rivera-Santiago et al. (187). Band 3 is also an excellent system for applying computational methods, which have been used to study conformational effects of mutations (417); protein-protein and lipid-protein interactions (178, 451, 509); transport-related conformational changes (461, 510); subcellular diffusion (353, 482, 484); and integrative modeling of the interplay between transport and carbonic anhydrase activity (482, 511, 512). As computational methods improve, it may become possible to model substrate binding to band 3 and gain insights into the nature of the transition state. It should also become possible to simulate, simultaneously, all or most of the processes shown in Fig. 9. The combination of computational approaches and the vast store of existing (and future!) experimental data has the potential for producing a genuine understanding of both the molecular mechanism and the integrative physiology of band 3-mediated anion transport. We are much closer than we were a few years ago, but there is still more to do.

## GRANTS

My work on band 3 was supported for many years by NIH Grant R01 GM026861 and institutional support from the University of Iowa, the University of Texas Medical Branch, and the University of Arkansas for Medical Sciences.

## DISCLOSURES

No conflicts of interest, financial or otherwise, are declared by the author.

## AUTHOR CONTRIBUTIONS

M.L.J. prepared figures; drafted manuscript; edited and revised manuscript; and approved final version of manuscript.
